# Stratified Bacterial and Archaeal Community in Mangrove and Intertidal Wetland Mudflats Revealed by High Throughput 16S rRNA Gene Sequencing

**DOI:** 10.3389/fmicb.2017.02148

**Published:** 2017-11-02

**Authors:** Zhichao Zhou, Han Meng, Yang Liu, Ji-Dong Gu, Meng Li

**Affiliations:** ^1^Laboratory of Environmental Microbiology and Toxicology, School of Biological Sciences, University of Hong Kong, Pokfulam, Hong Kong; ^2^Institute for Advanced Study, Shenzhen University, Shenzhen, China

**Keywords:** stratification, microbial community, mangrove wetlands, sediment profiles, pH, seasonality

## Abstract

The stratified distribution of bacterial and archaeal communities has been detected in many sediment profiles from various natural environments. A better understanding of microbial composition and diversity pattern in coastal mangrove wetlands in relation to physicochemical and spatial-temporal influences could provide more insights into the ecological functions of microbes in coastal wetlands. In this study, seasonal variations of microbial communities within sediment profiles from two sediment types (mangrove forest and intertidal mudflats) at three sampling locations in coastal Mai Po wetland were characterized using MiSeq high throughput sequencing and 16S rRNA quantitative PCR methods. Bacterial 16S rRNA gene abundance showed clear decreasing trends with increasing depth for all sites, seasonality and sediment types. There is a weak seasonal dynamic of bacterial and archaeal community abundance in both seasons. Seasonality imposed more influence on the beta diversity pattern of bacterial community than archaeal community. The five most abundant phyla within bacterial and archaeal community remain stable between two distinctive seasons. Sediment depth and seasonality are the most influential factors affecting bacterial community composition and diversity. The pH is the most influential factor on shaping the archaeal community. Stratified distribution of bacterial community including aerobic and anaerobic bacterial taxa is largely represented in the surface layers and the subsurface layers, respectively. For archaeal stratification*, Thaumarchaeota* Marine Group I is the dominant member in surface sediments while *Bathyarchaeota* and MBG-B dominate in subsurface sediments. Such stratified distribution patterns are irrespective of sediment types, sampling locations or seasonality, but significantly correlated to the sediment depth, which might be shaped by oxygen availability and the distribution of other terminal electron accepters along the depth profile.

## Introduction

Coastal mangrove forest is one of the most important integrated ecosystems mainly distributed in the tropical or subtropical coastline and estuarine areas with significant ecological functions, such as filtering and reducing dissolved and particulate nutrients, serving as a sink for carbon, nitrogen and phosphorus, as well as retaining heavy metals from adjacent land and fluvial imports (Zheng et al., 2000; Reef et al., 2010; Alongi, 2014; Sanders et al., 2014). The mangrove forests, characterized as highly productive ecosystems, contribute to 10–15% global coast sediment carbon storage, and provide nutrients and habitats for microorganisms, micro-/marco-fauna, and migratory birds (Yan et al., 2006; Alongi, 2014; Bhattacharyya et al., 2015). The microorganisms inhabiting mangrove forests have important roles in facilitating carbon, sulfate, nitrogen and phosphorus cycles as well as promoting plant growth by generating phytohormone and siderophore (Holguin et al., 2001). Increasing researches are focused on the distribution pattern and potential biogeochemical functions in mangrove ecosystems (Nedwell et al., 1994; Yan et al., 2006; Sahoo and Dhal, 2009; Wang et al., 2012; Jiang et al., 2013; Bhattacharyya et al., 2015). Linking microbial distribution and diversity pattern to environmental factors would allow better understanding of ecosystem functions and biogeochemical processes within the local wetland, thus facilitating the wetland management and sustainable development (Sims et al., 2013; Ansola et al., 2014; Arroyo et al., 2015).

Mai Po Nature Reserve locates at the northwestern coastal region of Hong Kong, facing Deep Bay, and it is influenced by discharges from Shenzhen River and Pearl River. Mai Po Nature Reserve is the largest wetland in Hong Kong, listed as a Ramsar Site for its importance of environmental reservation and management (Liang and Wong, 2003). Its ecological importance is renowned for offering conservation of micro-/macro-fauna, migratory birds, and mangrove forests which mainly dominated by *Kandelia obovata* and *Avicennia marina* (Lee, 1999; Jia et al., 2014). It contains several man-made and natural eco-niches, including, fresh water ponds, reedbeds, *Gei Wai* (semi-enclosed shrimp ponds), mangrove forests and fringe intertidal mudflats. The rapid urbanization and industrialization coupled with fast population augmentation around the Pearl River Delta Zone have taken place during the economic boost over the last several decades in South China (Lee et al., 2006). It has imposed large environmental stress, such as heavy metals, organic pollutions and eutrophication impacts on the estuarine regions, as well as western coastal water of Hong Kong (Zheng et al., 2000; Cheung et al., 2003; Liang and Wong, 2003). The catchments of local rivers (such as Shan Pui River and Kam Tin River) for livestock (such as poultry and animal husbandry) and domestic sewage in northwestern New Territories have also deteriorated the ecological conditions (Lau and Chu, 1999; Lee, 1999).

The recent research found the spatial distribution of bacterial communities toward the horizontal and vertical gradients of freshwater lakeside sediments (Ding et al., 2015). The variable biogeochemical zonation along the depth profile involves the mineralization process, and could largely determine bacterial community distribution patterns (Wilms et al., 2006; Canfield and Thamdrup, 2009; O'sullivan et al., 2013; Ding et al., 2015). Many studies also revealed many influential factors, such as wetland types (constructed or natural wetland), water contents, organic matters, total Kjeldahl Nitrogen (TKN), chemical oxygen demand (COD), inorganic nitrogen (${\text{NH}}_{4}^{+}$ and ${\text{NO}}_{3}^{-}$) and pH are determinants of soil bacterial community composition and structure (Fierer et al., 2003; Drenovsky et al., 2004; Ansola et al., 2014; Ligi et al., 2014; Arroyo et al., 2015; Ding et al., 2015). Meanwhile, temporal variables of seasonality could also influence the bacterial diversity pattern, and community composition and abundance of certain functional groups (Wang et al., 2013; Lu et al., 2016). Stratified distribution of archaeal community in estuarine sediment depth profiles has been well documented regarding to compositional patterns between surface and subsurface sediments (Webster et al., 2010; Jiang et al., 2011; Li et al., 2012). Recently, comparison of the bacterial community difference among mangrove wetlands, marine sediments and freshwater sediments was reported (Wang et al., 2012). The bacterial community composition and diversity between inner and outer mangrove forest sediments revealed spatial variation among sediment types (Jiang et al., 2013). However, most of the above researches have seldom paid an integrative view on microbial distribution and diversity patterns toward spatial and temporal scales, and statistically summarize the pattern reflected in a whole ecosystem. Bacterial and archaeal community distributing pattern toward depth profiles, sediment types, seasonality, and sampling locations in coastal mangrove wetlands still need to be comprehensively analyzed. The potential linkage between the physicochemical factors and microbial community remains elusive; it is fundamental to address the underlying mechanism for the formation of niche-specific microbial community structures and functions and their ecological roles in local biogeochemical processes (O'sullivan et al., 2013; Ansola et al., 2014; Ligi et al., 2014; Arroyo et al., 2015).

The rapid development of high throughput sequencing techniques and related bioinformatic approaches have allowed the exploration of microbial community with sufficient sequence coverage and equal sampling scale, which enables robust and comprehensive assessment on reflecting microbial distribution pattern (Dowd et al., 2008; Schloss et al., 2009; Caporaso et al., 2010, 2012; Angiuoli et al., 2011; Quast et al., 2013; Cole et al., 2014). In this study, the Illumina MiSeq based on 16S rRNA genes high throughput sequencing method was applied to study the temporal and spatial variation influence, including vertical distribution pattern, sampling locations and sediment types together with the seasonality influence, on the microbial community structure in Mai Po wetland. The microbial distribution patterns within depth profiles from two sediment types, mangrove forest (MG) and intertidal mudflats (TF), were compared and analyzed. It is aimed to characterize stratified microbial communities and their composition and diversity patterns in relation to the physicochemical factors.

## Materials and methods

### Sampling, physicochemical parameter measurement and DNA isolation

**Figure 1:**
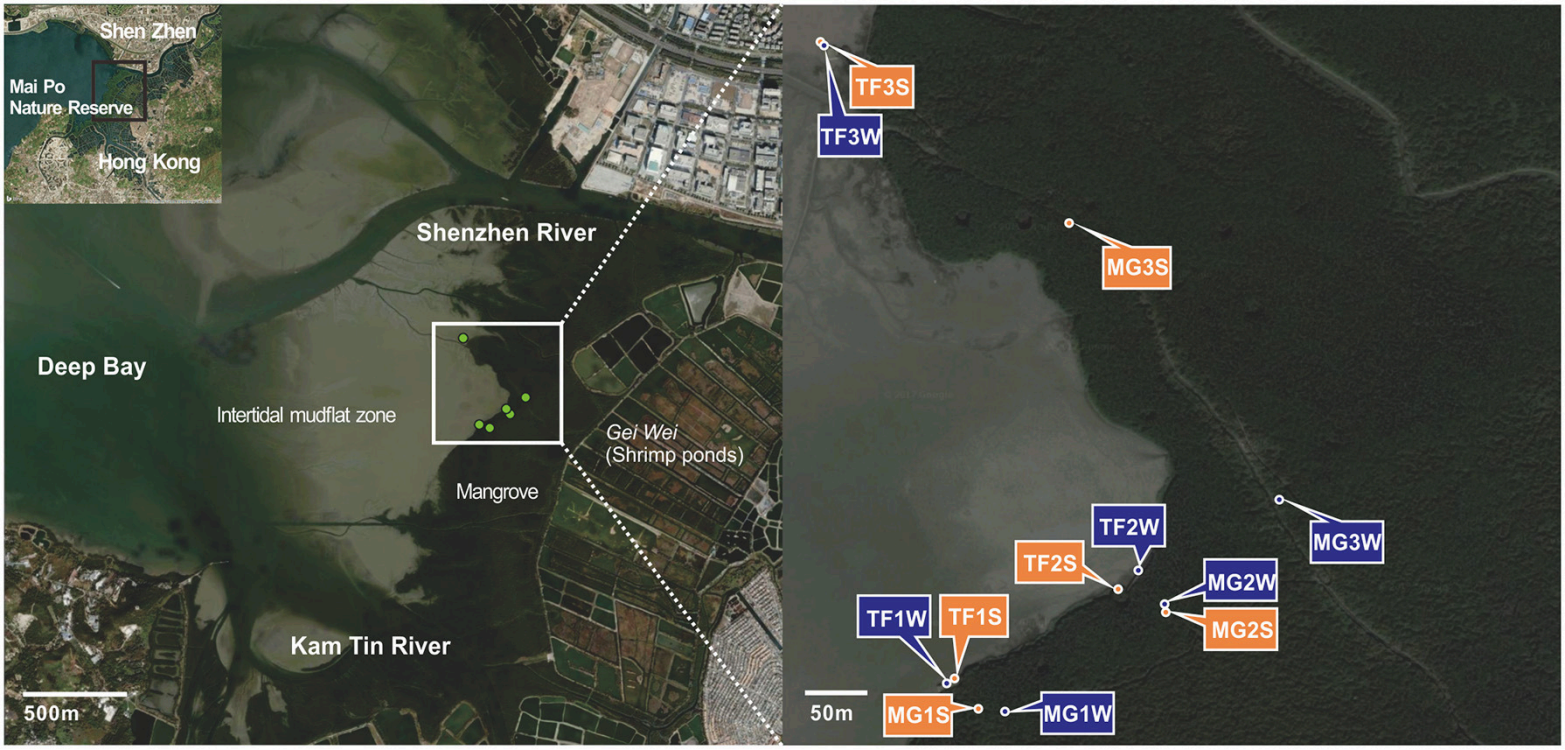
Satellite map depicting Mai Po area and sampling locations based on ArcGIS with Bing Aerial layout and Google Map.

Samples were taken in Mai Po Nature Reserve, a coastal wetland located at Shenzhen River estuary and facing the Inner Deep Bay (Shenzhen Bay) (Figure 1). Three sites were chosen from mangrove forest (MG1-3) and another three were from intertidal mudflats (TF1-3). At each site of mangrove forest, four layers of sediment were collected in winter and three layers were collected in summer. At each site of intertidal mudflats, two layers of sediments were collected in both two seasons (Table 1). Bulk sediments were sealed into plastic bags immediately after collection and stored in pre-cold sampling box immediately, then transported to laboratory. For each sample, 5 g of wet sediments were used for physicochemical parameter measurement and the remaining was stored in −20°C refrigerator for further DNA isolation.

**Table 1:** Physicochemical parameters of samples collected from Mai Po Nature Reserve.

**Samples**	**Sampling position**	**Depth (cm)**	**pH**	**Redox (mV)**	**Water content (%)**	**${\text{NH}}_{4}^{+}$ (μmol/kg dry soil)**	**${\text{NO}}_{2}^{-}$(μmol/kg dry soil)**	**${\text{NO}}_{2}^{-}$+${\text{NO}}_{3}^{-}$(μmol/kg dry soil)**	**Organic matters (%)**
MG1WinA	22°29.665′N, 114°01.742′E	0–2	7.07	300	70.7	57.26	2.08	106.29	11.2
MG1WinB		10–15	6.38	296	58.9	91.28	1.05	3.86	13.3
MG1WinC		20–25	7.21	251	58.6	141.02	0.53	1.56	22.9
MG1WinD		40–45	7.60	219	59.4	95.87	0.57	1.36	12.3
MG2WinA	22°29.709′N, 114°01.812′E	0–2	7.11	264	55.6	29.60	0.63	15.82	11.2
MG2WinB		10–15	6.41	259	54.8	27.03	trace	1.37	11.1
MG2WinC		20–25	6.84	235	56.4	58.97	0.14	1.12	10.6
MG2WinD		40–45	7.13	217	52.6	51.98	0.21	0.82	10.1
MG3WinA	22°29.762′N, 114°01.866′E	0–2	6.41	266	54.0	14.68	trace	8.81	11.2
MG3WinB		10–15	6.27	209	57.0	142.83	trace	0.49	12.7
MG3WinC		20–25	6.79	202	51.4	156.43	trace	1.36	10.8
MG3WinD		40–45	7.40	209	50.4	78.16	0.12	0.41	9.7
TF1WinA	22°29.676′N, 114°01.706′E	0–5	7.06	217	56.2	55.55	4.43	23.53	10.0
TF1WinB		13–16	7.68	212	55.2	312.56	1.58	3.72	8.2
TF2WinA	22°29.726′N, 114°01.799′E	0–5	7.45	−63.6	69.8	15.84	2.19	5.50	12.1
TF2WinB		13–16	7.77	58	58.8	214.46	1.87	2.56	9.6
TF3WinA	22°29.951′N, 114°01.651′E	0–5	7.02	−42.8	65.0	7.10	1.01	6.24	10.1
TF3WinB		13–16	7.52	106.6	57.2	250.75	1.32	2.25	8.5
MG1SumA	22°29.665′N, 114°01.726′E	0–2	6.68	398	62.0	127.41	1.22	14.67	14.5
MG1SumB		10–15	7.58	171	57.5	348.06	1.20	7.13	13.1
MG1SumC		20–25	7.90	95	53.0	307.89	0.88	6.38	11.4
MG2SumA	22°29.704′N, 114°01.814′E	0–2	5.82	348	53.6	25.52	0.50	18.56	10.7
MG2SumB		10–15	6.87	291	51.4	22.21	0.73	9.13	14.4
MG2SumC		20–25	6.84	244	57.3	20.93	0.74	7.89	12.6
MG3SumA	22°29.875′N, 114°01.767′E	0–2	6.60	312	54.8	26.97	0.76	22.25	12.8
MG3SumB		10–15	7.31	298	52.4	15.96	1.75	11.19	12.9
MG3SumC		20–25	7.32	241	46.4	22.41	0.50	9.10	11.1
TF1SumA	22°29.679′N, 114°01.709′E	0–5	7.10	80	61.8	107.74	2.82	27.03	10.0
TF1SumB		13–16	7.86	179	54.8	262.91	0.85	7.43	8.9
TF2SumA	22°29.718′N, 114°01.786′E	0–5	7.38	−35	66.4	187.17	6.60	43.17	10.5
TF2SumB		13–16	8.17	65	55.8	368.63	0.83	4.39	9.7
TF3SumA	22°29.949′N, 114°01.656′E	0–5	7.31	111	60.1	127.18	2.64	22.81	10.3
TF3SumB		13–16	7.81	170	56.3	334.54	2.00	9.84	8.1

*MG, stands for mangrove forest; TF, stands for intertidal mudflats. Two groups of samples were sampled from winter and summer. Depth A, B, C, D were labeled as the last letter in sample names from surface to subsurface layers*.

The physicochemical parameters, including pH, redox potential, water content, ${\text{NH}}_{4}^{+}$, ${\text{NO}}_{2}^{-}$, and ${\text{NO}}_{3}^{-}$ concentrations, organic matter content, were measured using procedures as previously reported (Cao et al., 2012; Li et al., 2013). For DNA isolation, 0.25 g of wet sediments from each sample were used for metagenomic DNA isolation by PowerSoil® DNA Isolation Kit (MO BIO) and the procedures were those of the manufacturer's. The water contents of sediment samples were determined after heating in 105°C oven over night and DNA concentration in dry sediment was used in the final expression of results.

### MiSeq based 16S rRNA gene high throughput sequencing

In order to acquire specific archaeal 16S rRNA gene libraries from all samples, a nested PCR procedure was adopted. Long fragment targeting primer pair 21F/958R was used for the first step (DeLong, 1992), subsequently, successfully obtained and purified PCR products were applied as DNA templates for the second PCR step by using Arch349F/Arch806R (Takai and Horikoshi, 2000) as the primer pair (12 nt unique barcode was added to 5′ of Arch349F as indexing)(Caporaso et al., 2012). The PCR reaction mixture contained: 5 μl of 5 × GoTaq buffer (Promega), 5 nmol of dNTPs, 62.5 nmol of Mg^2+^, 10 μg of BSA (10 mg/ml, Roche), 0.5 μl of forward and reverse primer (20 μM), 1 μl of DNA template (0–25 ng/μl) and 0.2 μl of GoTaq polymerase (5 U/μl, Promega) and ddH_2_O to make up a total volume to 25 μl. The PCR thermocycling setting for the first PCR step (21F/958R) was as following: firstly pre-heating 95°C for 5 min; then 33 cycles of 95°C 30 s, 52°C 30 s and 72°C 90 s; finally 72°C for 10 min and 4°C for 2 min. The PCR thermocycling setting for the second PCR step (Arch349F/Arch806R) was as following: firstly preheating 95°C for 5 min; then 15 cycles of 95°C 30 s, 50°C 30 s and 72°C 40 s; finally 72°C for 10 min and 4°C for 2 min. For microbial 16S rRNA gene library construction, the primer pair 515F/909F was applied to cover the 16S hypervariable V4-V5 regions (12 nt unique barcode was added to 5′ of 515F as indexing) (Wang and Qian, 2009; Caporaso et al., 2012). The PCR thermocycling setting was as following: firstly pre-heating 95°C for 5 min; then 33 cycles of 95°C 30 s, 52°C 30 s and 72°C 90 s; finally 72°C for 10 min and 4°C for 2 min. The mixture for microbial 16S rRNA gene PCR was similar to that of archaeal 16S rRNA gene PCR, except for replacing each forward and reverse primer volume to 1 μl (20 μM). Every PCR reaction was conducted separately for duplicates and PCR products were pooled into one to obtain enough quantity and compromise PCR bias between batches. Then, each obtained PCR product was subjected to electrophoresis using 1% agarose gel. Clear bands with proper length were cut out and purified by illustra GFX PCR DNA and Gel Band Purification Kit (GE Healthcare). The concentrations of purified amplicons were measured by Nanodrop. Purified amplicons from all PCR libraries were pooled into one with the concentration to achieve 100 ng for individual sample, and then subjected to MiSeq for high throughput sequencing. The sequencing samples were prepared using TruSeq DNA kit according to manufacturer's instruction. The purified library was diluted, denatured, re-diluted, and mixed with PhiX (equal to 30% of the final DNA amount) as described in the Illumina library preparation protocols, and then applied to an Illumina MiSeq system for sequencing with the reagent kit v2 (2 × 250 bp) or v3 (2 × 300 bp) as described in the manufacturer's manual.

### MiSeq sequencing data process by QIIME

Firstly, two pair end sequencing data were merged into one using FLASH-1.2.8, then, fastx-toolkit was applied to split the merged data from one run into individual samples according to the attached barcodes (Magoc and Salzberg, 2011). Finally, each library was assigned by using QIIME software command “split_libraries” according to the corresponding barcode map file (Caporaso et al., 2012). Filtering criterion was set as “-s 15 -k -a 6 -r -l 150 -b 12 -M 5 -e 0.” The chimera check was conducted by USEARCH software using QIIME compatible SILVA 119 release SSURef database “rdp_gold” fasta file as the reference (Edgar, 2010; Caporaso et al., 2012). After screening out the chimeric sequences from each library, sequences from all libraries were combined into one and subjected to OTU picking step, by applying the command “pick_open_reference_otus.py” with QIIME compatible SILVA 119 SSURef database (Silva_119_rep_set97.fna) as the reference. Open reference OTU picking method could effectively reduce the final OTU numbers by firstly clustering against reference sequences, picking up sequences clustering around references as centroids with 0.97 similarity, then, re-clustering remaining sequences which could not be assigned to reference OTUs by *de novo* method. Singletons were filtered out to exclude potential sequencing errors. Taxonomy assignment was conducted by BLAST method, applying QIIME compatible SILVA 119 SSURef database as the reference (newly defined archaeal taxa were manually added and recurated). The matrix file “biom” was made by adding the taxonomic referring information into OTU table. Archaeal “biom” file was made by exclusively filtering non-archaeal sequences from OTU table obtained from archaeal 16S rRNA gene MiSeq sequencing libraries. Similarly, bacterial “biom” file was made from microbial 16S rRNA gene MiSeq sequencing libraries by filtering non-bacterial sequences. Microbial “biom” file was made without any filtering. Table summary information including the sequence numbers in each library could be reflected by “summarize-table” command. The smallest library's size number was used to subsample all libraries by using “multiple_rarefactions_even_depth.py” command. Compositional profiles based on taxonomical information were depicted by “summarize_taxa_through_plots.py” using map file to index each library. Aligning was conducted by PyNAST method using “core_Silva119_alignment.fna” from QIIME compatible SILVA 119 SSURef database as the reference with 60% similarity criterion. Alignment filtering was conducted independently without lanemask file, with entropy threshold as 0.1 and gap filter threshold as 0.9. Phylogenetic tree was constructed by default. Then, alpha diversity calling was conducted with “biom” files after rarefaction. Alpha diversity indices including Shannon, PD whole tree, Chao1, observed species, Good's coverage, and Simpson values were generated accordingly. Beta diversity procedure was conducted by applying both unweighted, weighted UniFrac matrix and non-phylogenetic Bray-Curtis matrix methods.

### Diversity and statistical analysis

Stack bar charts for delineating taxonomic profiles of bacterial, archaeal and microbial community of all libraries were originated from QIIME results, and visualized by Origin8 and CorelDraw. Pearson correlation analysis on reflecting the potential correlation relationship between physicochemical parameters and diversity, abundance property of bacterial, archaeal and microbial communities was conducted in GraphPad Prism (Motulsky, 1999). Correlation coefficient matrix was generated by two-tailed *p*-value statistics. Statistical analysis of alpha diversity was conducted in IBM SPSS software by one-way ANOVA (with Dunnett T3 *post hoc* test) and unpaired *t*-test (SPSS Inc., Chicago). Mantel Test analysis implemented in QIIME was conducted to analyze the correlation relationship between physicochemical parameters and UniFrac and Bray-Curtis distance matrices from beta diversity analysis. Multivariate regression tree (MRT) was constructed using the R package mvpart, processed by normalized compositional abundance at the class level. The selected trees with 7-split tree size were depicted to reflect the explanatory effect of environmental variables on the community structures (Oksanen et al., 2007; Therneau et al., 2012). Principal Coordinate Analysis (PCoA) on depicting the dissimilar relationship of samples was conducted in terms of sediment depth and seasonality categories, based on both phylogenetic and non-phylogenetic distance matrix methods. To test whether the distribution of sample plots in PCoA analysis was statistically significant, both of adnois and anosim methods within “compare_categories” command in QIIME were applied. LDA Effect Size (LEfSe) analysis was applied on identifying biomarker taxa, which are significantly associated within certain sample categories (Segata et al., 2011). CANOCO 5.0 was used to conduct Redundancy Analysis (RDA) for depicting the explaining effect of environmental factors on the ordination of samples and their compositional taxa.

### Quantification of abundance of bacteria and archaea

To quantify the 16S rRNA gene copy number of bacteria and archaea in each sample, quantitative PCR measurement and statistical analysis were employed by StepOnePlus Real-Time PCR System instrument (Applied Biosystems). Primer pair Arch349F/958R was used to detect the archaeal 16S rRNA gene with annealing temperature of 52°C, primer pair Bac331F/Bac797R was used to detect the bacterial 16S rRNA gene with annealing temperature of 50°C (Nadkarni et al., 2002). The rest denaturing and elongation time and temperature were set according to the manufacturer's instruction. The 15μl qPCR system contained the following reagents: 1 μl of DNA template (10-fold-diluted to avoid interference of humic acids), 7.5 μl of Premix (FastStart Universal SYBR Green Master, Roche), 12 μg of BSA (100 mg/ml, Roche), 0.375 μl of forward and reverse primer (20 μM) for archaeal qPCR, alternatively 0.15 μl of forward and reverse primer (20 μM) for bacterial qPCR and ddH_2_O (make up final volume to 15 μl).

One positive ligated plasmid of PMD-18T with gene fragments from previously prepared PCR products was used to make the successive 10-fold dilution series for generating standard curves for archaeal and bacterial qPCR. Copy numbers of standard plasmid dilution were calculated by firstly measuring the DNA concentration by Nanodrop and then applied into the equation: Abundance of gene copy number/μl = (amount/μl × 6.022 × 10^23^)/(length × 1 × 10^9^ × 660). Results deviated unreasonably from values in the replicate groups were omitted and undetermined results were deleted. Final adjusted standard curve properties were as following, archaea: *r*^2^ = 0.998–0.999, Eff% = 64.447−69.356; bacteria: *r*^2^ = 0.994−0.997, Eff% = 91.226−92.464.

### Sequencing result deposition

Raw MiSeq sequencing data for 33 archaeal 16S rRNA gene libraries from Mai Po wetland were deposited in EMBL-EBI ENA database with accession no. PRJEB12429. Raw MiSeq sequencing data for 33 microbial 16S rRNA gene libraries from Mai Po wetland were deposited in EMBL-EBI ENA database with accession no. PRJEB12432.

## Results

### Physicochemical properties of sediment samples in winter and summer

Generally, patterns of different physicochemical properties of sediment depth profile of each sampling site were determined for sediment types at different seasons (Table 1). In winter samples, sediments of B layer at mangrove forest (MG) had the lowest pH values, and the redox potential and water content values generally showed a decreasing trend along the increase of sediment depth. The C layer of sediments showed the highest concentration of ${\text{NH}}_{4}^{+}$, and the upper layers (A and B layers) tended to acquire higher ${\text{NO}}_{2}^{-}$, Σ(${\text{NO}}_{3}^{-}$ + ${\text{NO}}_{2}^{-}$) concentration and organic matter content than the corresponding lower layers (D layers), except for the organic matter content value of MG1WinC, which was considerably higher than the others. In winter intertidal mudflat (TF) samples, pH, redox potential and ${\text{NH}}_{4}^{+}$ were lower in the surface layers than in the subsurface layers. While, in terms of water content, ${\text{NO}}_{2}^{-}$, Σ(${\text{NO}}_{3}^{-}$ + ${\text{NO}}_{2}^{-}$), and organic matter content, higher values were observed in the surface layers than that in subsurface, with a few exceptions, such as redox potential values of TF1WinA and TF1WinB pair, and ${\text{NO}}_{2}^{-}$ of TF3WinA and TF3WinB pair. In summer MG samples, pH value showed a clear increasing trend along the increase of sediment depth, while a decreasing trend for redox potential value, water content and Σ(${\text{NO}}_{3}^{-}$ + ${\text{NO}}_{2}^{-}$) was found, except for the water content of MG2SumC sample. The ${\text{NH}}_{4}^{+}$ of samples from MG site 1 depth profile showed a considerable elevation than the site 2 and 3. In summer TF samples, patterns of depth profile at individual sampling sites were identical with those of winter TF samples. In addition, value ranges of all the parameters of summer TF samples were also close to those of winter TF samples.

### Abundance of archaea and bacteria

**Figure 2:**
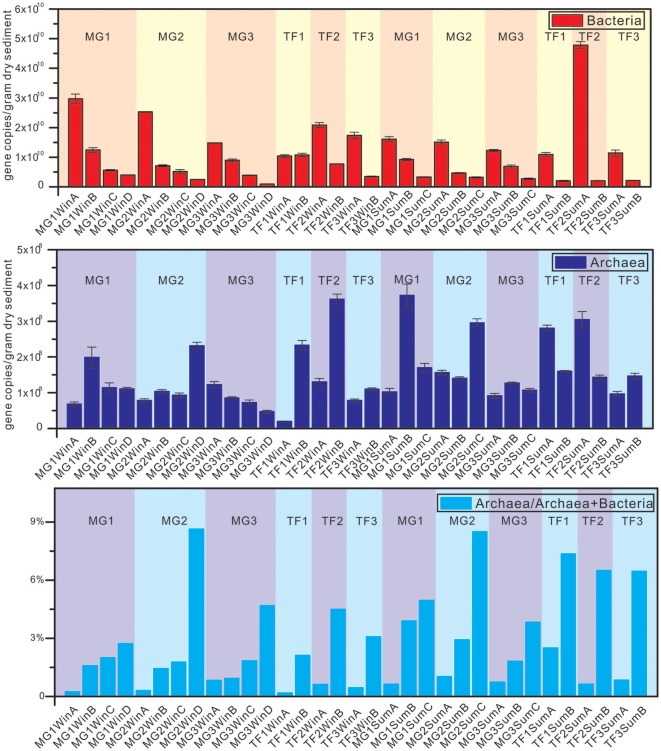
Quantitative PCR results on the abundance of bacterial and archaeal 16S rRNA genes of sediment samples from mangrove forest (MG) and intertidal mudflat (TF). The ratio of archaeal 16S rRNA genes over total archaeal and bacterial 16S rRNA genes reflects the archaeal fraction in the total microbial community. The unit of vertical axis is copy numbers/gram dry sediments.

Quantitative measurement of bacterial and archaeal community abundance was based on the 16S rRNA gene abundance (Figure 2, Supplementary Table S1). The bacterial community abundance of MG sediments showed a clear decreasing tendency from the surface layers down to the subsurface layers at all three sampling sites, in both winter and summer. A similar phenomenon was also evident that bacterial abundances in surface layers were always higher than the corresponding subsurface layers among TF sediment samples, irrespective of sampling sites and seasonality, except for TF1, where both surface and subsurface sediment samples had similar bacterial 16S rRNA gene abundances.

For archaeal community abundance, a different distribution pattern was identified. TF sample groups in winter showed an opposite distribution pattern from bacteria, in that lower archaeal abundance in the surface samples was detected than the corresponding subsurface samples. However, the relative abundance of archaea within the total microbial community quantified by 16S rRNA gene copy numbers represented a consistent trend opposite of the variation of bacterial community abundance in sediment depth profiles. Regardless of sampling sites, sediment types and seasons, the abundance of archaea always increased with the sediment depth, ranging from the lowest 0.19% (TF1WinA) to the highest 8.63% (MG2WinD) of the total microbial community.

The observed bacterial 16S rRNA gene abundance ranged from 9.6 × 10^8^ to 2.97 × 10^10^ and 2.02 × 10^9^ to 4.78 × 10^10^ gene copies/gram dry sediment in winter and summer, while the observed archaeal 16S rRNA gene abundance ranged from 1.96 × 10^7^ to 3.62 × 10^8^ and 9.17 × 10^7^ to 3.72 × 10^10^ gene copies/gram dry sediment in winter and summer, respectively. Although the range of 16S rRNA gene abundance was wider in summer than in winter, there was no significant difference in either bacterial or archaeal 16S rRNA gene abundance between summer and winter based on unpaired *t*-test (*p* > 0.05). Detailed statistical results of bacterial and archaeal 16S rRNA gene abundance are listed in Supplementary Table S1.

### Compositional summary of bacterial and archaeal community

**Figure 3:**
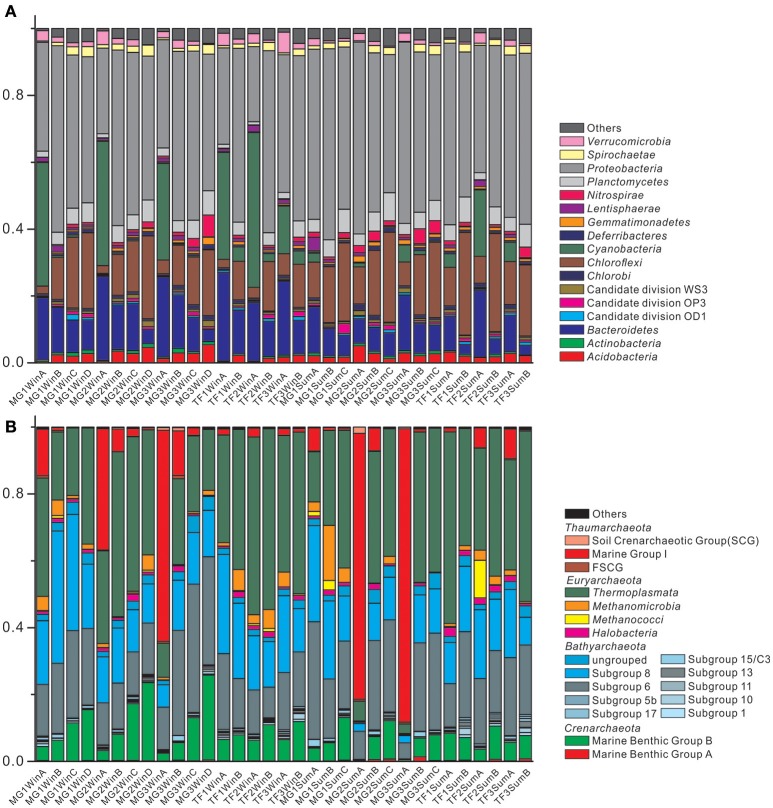
Bar chart of taxonomic profiles of **(A)** bacterial community at phylum level with abundance >0.5%, and **(B)** archaeal community at class level with abundance >0.1%. The remaining groups with lower abundance are classified into “Others.”

The composition of bacterial communities of 33 sediment samples from Mai Po wetland was shown in Figure 3A. The bar chart of taxonomic composition is shown for the phylum level bacterial taxa abundance, represented by average abundance among all samples >0.5%. The most abundant five bacterial phyla are: *Proteobacteria* (45.6%), *Chloroflexi* (14.7%), *Bacteroidetes* (12.0%), *Cyanobacteria* (7.6%), and *Planctomycetes* (4.5%) (Supplementary Table S2). In terms of different sediment types, the most abundant five phyla ranking remained stable. Among the subgroups of *Proteobacteria, Deltaproteobacteria* (21.9%) and *Gammaproteobacteria* (15.0%) were the most abundant components, while *Betaproteobacteria, Epsilonproteobacteria*, and *Alphaproteobacteria* were minor groups, contributing a small proportion (2.7, 2.7 and 2.6%) in the total bacterial community. For the second most abundant phylum, *Chloroflexi*, it was mainly comprised of two classes: *Anaerolineae* (9.1%) and *Dehalococcoidia* (4.4%). *Bacteroidetes* was composed of *Flavobacteriia* (4.4%), *Cytophagia* (1.8%), *Sphingobacteriia* (1.3%), BD2-2 (1.2%), SB-1 (0.7%), and SB-5 (0.7%). While *Cyanobacteria* was mainly represented by the subordinate class *Chloroplast* (7.5%), *Planctomycetes* was mainly represented by the subordinate class *Phycisphaerae* (2.3%) and *Planctomycetacia* (1.1%). Furthermore, *Cyanobacteria* was regularly enriched in the surface sediments, irrespective of sediment types, but mainly represented in winter samples. *Bacteroidetes* and *Chloroflexi* showed an opposite distribution pattern for their relative abundances in the surface and subsurface layers, regardless of sediment types and seasons. *Bacteroidetes* abundance fraction decreased along the sediment depth at the individual sampling site, while *Chloroflexi* abundance fraction increased along the sediment depth at the individual sampling site.

The composition of archaeal communities of 33 sediment samples from Mai Po wetland was shown in Figure 3B. The bar chart of archaeal community composition was represented by taxa abundance at the class level with an average abundance >0.1%. The most abundant five classes among all the archaeal communities were *Thermoplasmata* (34.2%), *Bathyarchaeota* Subgroup 6 (20.1%), and Subgroup 8 (16.8%), Marine Group I (10.5%) and Marine Benthic Group B (MBG-B) (8.4%) (Supplementary Table S3). They occupied almost 90% of the average abundance in the total archaeal community. There were still other *Bathyarchaeota* subgroups, such as Subgroup 1, 5b, 10, 11, 13, 15/C3, 17, and the ungrouped *Bathyarchaeota*. The total *Bathyarchaeota* contributed to as high as 41.8% of the average abundance in the total archaeal community. The other uncultured group of *Crenarchaeota*, Marine Benthic Group A (MBG-A), could only account for 0.3% of the average abundance of the total archaeal community. For the methanogenic *Euryarchaeota* class, *Methanomicrobia* and *Methanococci* accounted for 2.6% of the average abundance of the total archaeal community. MBG-B showed a regular distribution pattern of their abundances, increasing along the sediment depth at the individual sampling site, irrespective of sediment types and seasons. Moreover, Marine Group I (*Thaumarchaeota*) only showed their considerably higher proportion in surface samples than the corresponding subsurface samples at the individual sampling site, yet their contribution among surface samples could also vary greatly, ranging from as low as 1.1% in TF1SumA to as high as 87.8% in MG3SumA. Similarly, the order of the most abundant five classes remained unchanged, in terms of two sediment types.

### Alpha diversity of bacterial, archaeal, and microbial communities

Through alpha processing pipeline by QIIME software, alpha diversity indices, including PD whole tree, Chao1, Good's coverage, observed species, Shannon and Simpson indices, were calculated for individual bacterial, archaeal and microbial communities (Supplementary Table S4). The Good's coverage of bacterial, archaeal and microbial communities ranged within 66.1–84, 90.3–98.2, and 67.6–83.4%, respectively. The rarefaction curves of the above three communities indicated that the Good's coverage values of all samples achieved the plateau (data not shown), and further increasing of sequencing efforts will not significantly elevate the Good's coverage values. The statistical comparisons of these alpha diversity indices among sediment samples classified by layer depth, seasonality, sediment type and sampling site to find any significant differences of certain alpha diversity indices between each pair of sample categories or significant intergroup differences among all of the samples were conducted. It was revealed that, for all the bacterial communities, there were significant differences among the layer depth categories in terms of observed species, Shannon and Simpson indices. Depth A samples (the lowest layer) had the lowest value of the above alpha diversity indices. The unpaired *t*-test showed that the Shannon index in winter samples was lower than that in summer, for all the bacterial communities (*p* < 0.05). For archaeal communities, winter samples had higher Chao1 and observed species indices and lower Good's coverage value than those of summer (*p* < 0.05). Moreover, samples of intertidal mudflats had higher Simpson index than those of mangrove forest (*p* < 0.05), and higher PD whole tree, Chao1, observed species and Shannon indices and lower Good's coverage value than those of mangrove forest (*p* < 0.001). For the total bacterial and archaeal communities, there were significant differences among the four layer depths for the alpha diversity, including PD whole tree, Good's coverage, observed species, Shannon and Simpson indices. And the samples with the lowest layer depth (Depth A) had the lowest PD whole tree, observed species, Shannon and Simpson indices and highest Good's coverage values among these four categories (*p* < 0.05). The Simpson index of winter samples was significantly lower than that of summer samples (*p* < 0.05). Detailed comparison results of alpha diversity indices among different categorical divides are summarized in the Supplementary Table S4.

### Relationship of community properties and physicochemical parameters

In terms of the relationship between alpha diversity indices and physicochemical parameters, Shannon and Simpson indices of bacterial communities were positively correlated with sediment layer depth and negatively correlated with water content by the Pearson correlation analysis, with statistical support (*p* < 0.05) (Table 2). The observed species of bacterial communities were negatively correlated with Σ(${\text{NO}}_{3}^{-}$ + ${\text{NO}}_{2}^{-}$) (*p* < 0.05).

**Table 2:** Pearson correlation analysis between physicochemical parameters and 16S rRNA gene abundances together with alpha diversity indices of both bacterial and archaeal communities.

	**Depth**	**pH**	**Redox**	**Water content**	**Ammonium**	**Nitrite**	**Nitrate**	**Nitrate+ Nitrite**	**Organic matters**
**GENE ABUNDANCE**									
Bacterial 16S rRNA gene	−0.611^***^	−0.190	−0.207	0.667^***^	−0.200	0.626^***^	0.608^***^	0.633^***^	0.005
**ALPHA DIVERSITY (BACTERIA)**									
PD whole tree	0.223	−0.060	0.111	−0.295	0.254	−0.203	−0.328	−0.332	0.036
Chao1	0.129	−0.101	0.035	−0.149	0.196	−0.100	−0.262	−0.261	0.003
Good's coverage	−0.233	0.101	−0.127	0.274	−0.234	0.232	0.318	0.324	−0.049
observed species	0.325	−0.117	0.204	−0.366^*^	0.220	−0.301	−0.344	−0.354^*^	0.088
Shannon	0.399^*^	−0.112	0.259	−0.419^*^	0.208	−0.330	−0.309	−0.323	0.106
Simpson	0.371^*^	−0.065	0.249	−0.390^*^	0.245	−0.273	−0.192	−0.205	0.076
**GENE ABUNDANCE**									
Archaeal 16S rRNA gene	0.024	0.249	−0.290	0.125	0.386^*^	0.269	−0.092	−0.070	−0.067
**ALPHA DIVERSITY (ARCHAEA)**									
PD whole tree	0.021	0.516^**^	−0.648^***^	0.329	0.326	0.317	−0.081	−0.057	−0.445^**^
Chao1	0.008	0.432^*^	−0.611^***^	0.213	0.201	0.244	−0.100	−0.080	−0.506^**^
Good's coverage	−0.006	−0.453^**^	0.642^***^	−0.243	−0.221	−0.271	0.120	0.097	0.496^**^
Observed species	0.026	0.477^**^	−0.634^***^	0.280	0.249	0.292	−0.103	−0.079	−0.471^**^
Shannon	0.148	0.509^**^	−0.534^**^	0.327	0.369^*^	0.244	−0.047	−0.028	−0.334
Simpson	0.221	0.425^*^	−0.409^*^	0.287	0.362^*^	0.177	−0.068	−0.053	−0.165

“*”, “**”, “***” *Mean significantly different level between groups as “< 0.05”, “< 0.01”, “< 0.001”*.

For archaeal communities, pH was positively correlated with alpha diversity indices, including PD whole tree, Chao1, observed species, Shannon and Simpson indices, but negatively correlated with Good's coverage with statistical support; however, the correlation relationship between redox potential and these indices was the opposite trend. Moreover, ${\text{NH}}_{4}^{+}$ was positively correlated with Shannon and Simpson indices of archaeal community (*p* < 0.05), but organic matter content was negatively correlated with alpha diversity indices, including PD whole tree, Chao1 and observed species, and positively correlated with Good's coverage value (*p* < 0.01; Table 2).

**Figure 4:**
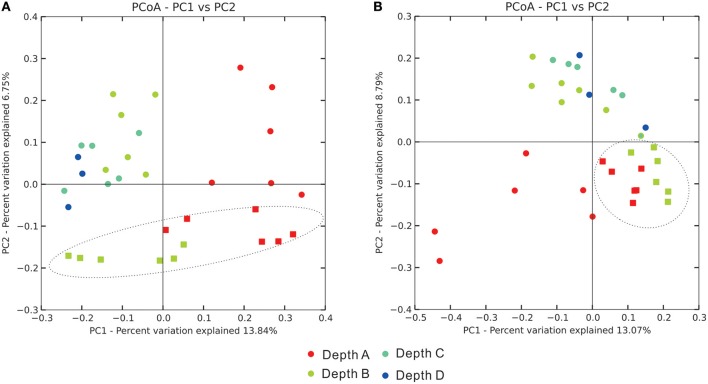
Principal Coordinate Analysis plots reflecting the dissimilar distance of **(A)** bacterial community and **(B)** archaeal community among samples. Dots stand for MG samples, squares stand for TF samples.

Principal Coordinate Analysis (PCoA) on depicting the dissimilar relationship of samples revealed that, in terms of sediment types, there were clear separations between these two categorical assemblages (sediment depths and sediment types) in both bacterial and archaeal community analyses (Figure 4). These patterns were further evidenced by statistical analyses of the distribution of community coordinates. Based on both the unweighted UniFrac and non-phylogenetic Bray-Curtis distance matrix method, it was also significant to differentiate these two categorical assemblages (sediment depths and sediment types) (Supplementary Table S5). In terms of the four layer depths, the differentiation patterns among these four categories in both bacterial and archaeal communities were significant by all distance matrix methods, especially for bacterial communities (*p* < 0.001; Figure 4, Supplementary Table S5). The other two categories (seasons and sampling sites) were also checked for identifying any influence on the beta diversity pattern of bacterial and archaeal communities. Seasonality could be influential on the beta diversity pattern of bacterial communities based on weighted UniFrac and Bray-Curtis distance matrix method, sampling sites could be influential on the beta diversity pattern of archaeal communities based on unweighted UniFrac and Bray-Curtis distance matrix method (Supplementary Table S5). Regarding to the total microbial communities, influences on their beta diversity pattern imposed by the four types of category were similar to those of bacterial communities.

Mantel Test was applied to delineate the correlation relationship between UniFrac and Bray-Curtis matrices of community composition and the distance matrix of physicochemical parameters (Supplementary Table S6). Layer depth, water content, ${\text{NO}}_{3}^{-}$ and Σ(${\text{NO}}_{3}^{-}$ + ${\text{NO}}_{2}^{-}$) were significantly correlated with bacterial community dissimilar distance matrix by both UniFrac and Bray-Curtis matrix method (*p* < 0.05). Redox potential was significantly correlated with bacterial community dissimilar distance matrix only by unweighted UniFrac and Bray-Curtis matrix method (*p* < 0.05). The pH was significantly correlated with bacterial community dissimilar distance matrix only by unweighted UniFrac matrix method (*p* < 0.05). However, only pH was significantly correlated with archaeal community dissimilar distance matrices by both UniFrac and Bray-Curtis matrix method (*p* < 0.01). Redox potential was significantly correlated with archaeal community dissimilar distance matrix only by unweighted UniFrac matrix method (*p* < 0.05).

**Figure 5:**
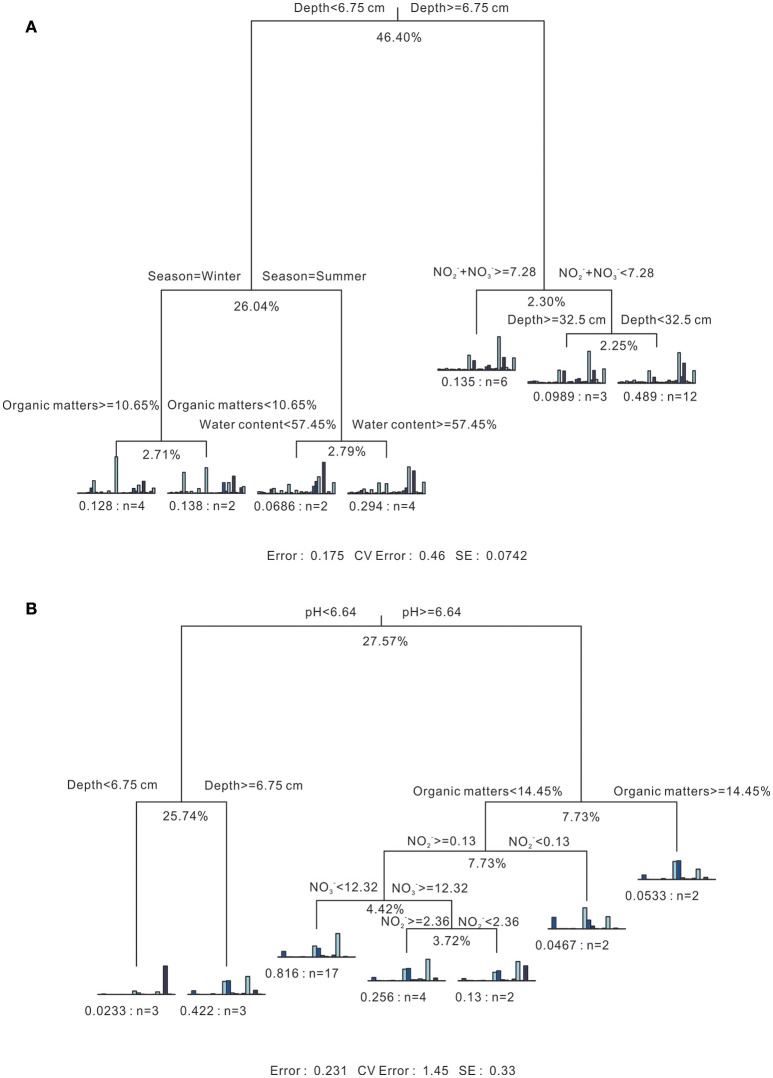
Multivariate regression tree analysis of the associated relationship of **(A)** bacterial and **(B)** archaeal communities and physicochemical parameters. For both figures, 7-split trees are visualized. Statistics information is listed under individual trees, including the residual error (the reciprocal of the *R*^2^ of the model), cross-validated error and standard error. Under each branch, a small bar plot is given to represent the normalized abundance of species. The corresponding residual error and sample numbers under each branch are also listed. Values under each split point indicate percentages of variance explained by the split (Complexity *R*^2^). Units for ${\text{NH}}_{4}^{+}$, ${\text{NO}}_{2}^{-}$ and ${\text{NO}}_{3}^{-}$ concentrations refer to Table 1.

As shown in the MRT (Figure 5A), sediment depth could account for 46.4% of the explained variance among all bacterial communities. The most discriminated groups under this splitting point were *Chloroplast* (*Cyanobacteria*) and *Deltaproteobacteria*, accounting for 43.64 and 27.13% of the explained deviance. Then, seasonality played a minor role in splitting the bacterial communities of surface layer sediments (Depth < 6.75 cm) into two branches, accounting for 26.04% of the total explained variance. The organic matter and water content could further divide the following winter and summer branches into four parts, with relatively small explaining effects. For the subsurface layer sediments (Depth ≥ 6.75 cm), Σ(${\text{NO}}_{3}^{-}$ + ${\text{NO}}_{2}^{-}$) could split the branch into two parts, one with the value higher than 7.28 μmol/kg dry soil, and the other with value smaller than 7.28 μmol/kg dry soil. Subsequently, the latter one could be further divided into two branches, including sediments from the deepest layer (Depth D), and the other above Depth D. The overall contribution of environmental variables on explaining the variance of whole bacterial communities was 82.5%.

In terms of the archaeal communities (Figure 5B), pH value could divide the MRT into two major branches, one contained samples with acidic pH condition, and the other one with pH value over than 6.64, which accounts for 27.57% of the explained variance. The most discriminated groups under this splitting point were *Thermoplasmata* and Marine Group I (*Thaumarchaeota*), accounting for 33.38 and 56.54% of the explained deviance. The former branch could be further divided into two by depth with the explaining effect of 25.74%. For the branch with pH higher than 6.64, five branches were formed by organic matters, ${\text{NO}}_{2}^{-}$ and ${\text{NO}}_{3}^{-}$ subsequently with decreasing explaining effects. The total contribution of environmental variables on explaining the variance of the whole archaeal communities was 76.9%.

### Differentiated distribution of bacterial and archaeal communities

**Figure 6:**
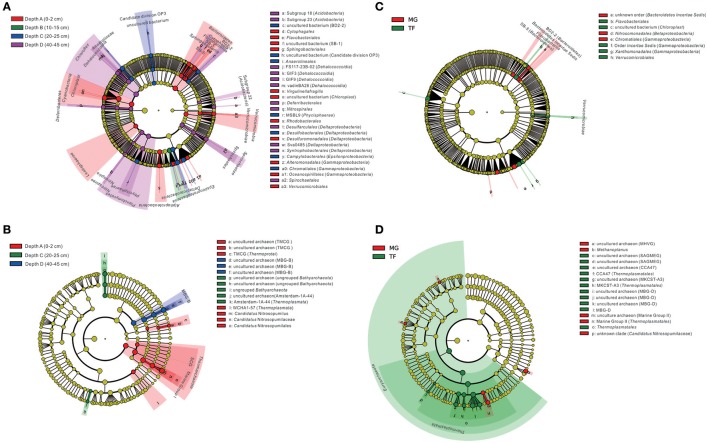
Cladogram based on LEfSe analysis results of bacterial **(A,C)** and archaeal **(B,D)** communities in terms of categories of sediment depth and sediment type (MG, mangrove forest; TF, intertidal mudflat). The taxonomic trees are depicted from phylum to order (for bacteria) and for phylum to genus (for archaea) in hierarchical structure from inside to outside. Biomarker taxonomic levels are labeled in color with at least 3.5 logarithmic LDA score. Pre-sample normalization is used to format the relative abundance. All-against-all strategy is used in the step of multi-class analysis.

As revealed by LEfSe analysis diagram for the depth layer (Figure 6A), bacterial taxa were differently enriched in each layer. Generally, the whole phylum *Bacteroidetes*, including most of the subordinate classes and orders shown in the diagram, was enriched in Depth A layer, except for Subgroup BD2-2, SB-5 enriched in Depth C layer. *Cyanobacteria, Lentisphaerae, Alphaproteobacteria, Verrucomicrobia* and their subordinate classes and orders shown in the diagram were enriched in Depth A layer. The order *Desulfuromonadales* (within *Deltaproteobacteria*), *Alteromonadales*, and *Oceanospirillales* (within *Gammaproteobacteria*) were also enriched in Depth A layer. *Deltaproteobacteria* as a whole was enriched in Depth B layer. Besides, Candidate division OP3, *Anaerolineae* (within *Chloroflexi*), *Anaerolineales* (within *Anaerolineae*), *Phycisphaerae* MSBL9 (within *Planctomycetes*), *Desulfobacterales* (within *Deltaproteobacteria*), *Epsilonproteobacteria, Campylobacterales* (within *Epsilonproteobacteria*), and *Chromatiales* (within *Gammaproteobacteria*) were enriched in Depth C layer. Three *Acidobacteria* subgroups, Subgroup 18, 22 and 23, phylum *Chloroflexi* as a whole, *Dehalococcoidia* (within *Chloroflexi*), *Deferribacteres, Nitrospirae, Spirochaetae*, and their subordinate orders shown in the diagram were enriched in D layer. *Planctomycetes* as a whole, *Phycisphaerae* (within *Planctomycetes*), *Desulfarculales*, Subgroup Sva048 and *Syntrophobacterales* (within *Deltaproteobacteria*) were also enriched in Depth D layer. In terms of differently distributed taxa in two types of sediment samples (Figure 6C), *Bacteroidetes Incertae Sedis* and Subgroup BD2-2, SB-5, *Nitrosomonadales* (within *Betaproteobacteria*) and *Chromatiales* (within *Gammaproteobacteria*) were enriched in mangrove covering field sediments, while, *Flavobacteriia* and order *Flavobacteriales* (within *Bacteroidetes*), *Chloroplast* uncultured bacterium (within *Cyanobacteria*), order *Incertae Sedis* and *Xanthomonadales* (within *Gammaproteobacteria*), *Verrucomicrobiae* and order *Verrucomicrobiales* (within *Verrucomicrobia*) were enriched in the intertidal mudflat sediments.

For archaeal taxa, *Thermoprotei* TMCG, *Thermoplasmata* WCHA1-57 and *Thaumarchaeota* (including Marine Group I and SCG) and their subordinate orders to genera shown in the diagram were enriched in Depth A layer (Figure 6B). Ungrouped *Bathyarchaeota* and their subordinate orders to genera shown in the diagram, together with Amsterdam-1A-44 uncultured clone (within *Thermoplasmata*) were enriched in Depth C layer. Marine Benthic Group B (MBG-B) and its subordinate orders to genera shown in the diagram were enriched in Depth D layer. In terms of the selective distribution of archaeal taxa in each sediment type (Figure 6D), Marine Group II (within *Thermoplasmata*), uncultured archaeon affiliated to Marine Group II, uncultured archaeon affiliated to Marine Hydrothermal Vent Group (MHVG), *Methanoplanus* and unknown clade of *Candidatus* Nitrosopumilaceae were enriched in mangrove forest sediments. While, phylum *Euryarchaeota*, class *Thermoplasmata*, order *Thermoplasmatales, Thermoplasmatales* CCA47, and uncultured archaeon affiliated to *Thermoplasmatales* CCA47, Marine Benthic Group D (MBG-D), uncultured archaeon affiliated to MBG-D, South African Goldmine Euryarchaeotic Group (SAGMEG) together with its subordinate and MKCST-A3 (within *Thermoplasmata*) together with its subordinates were enriched in the intertidal mudflat sediments.

## Discussion

### Physicochemical properties of Mai Po wetland samples

According to the rainfall and temperature record from Hong Kong Observatory, the mean temperature and total rainfall for the winter sampling day were 15.5°C and trace amount, and those for the summer sampling day were 28°C and 32.1 mm, respectively. The average values of mean temperature and total daily rainfall of 1 month before the winter sampling day were 16.9°C and trace amount and those of the same period before summer sampling day were 29°C and 16.6 mm, respectively. According to the *in situ* temperature measurement in this study, sediments from different layers and sampling sites represented very little variations on the sampling days from the general average temperature.

Nitrate concentration in Pearl River has increased for two to three times over the last three decades and the nitrogen load from Pearl River with seasonal fluctuation is one of the main sources of Hong Kong waters (Lee et al., 2006). The relatively high loading of nutrients and pollutions from discharges of Pearl River and Shenzhen River imposes impacts on Mai Po wetland more in summer than in winter (Lau and Chu, 1999; Lee, 1999; Lee et al., 2006). Mangroves benefit from the nutrient input in summer and their leaf litters will elevate the organic matter level in summer (Tam et al., 1998). The intertidal mudflats surface sediments showed a clear elevation of ${\text{NH}}_{4}^{+}$, ${\text{NO}}_{2}^{-}$ and Σ(${\text{NO}}_{3}^{-}$ + ${\text{NO}}_{2}^{-}$) in summer comparing to those in winter. It might result from the higher nutrient load from adjacent runoffs in summer. Fringe intertidal mudflats are more influenced by the surrounding seawater than the mangrove covering field (Zhou et al., 2010). As for the organic matter content, it might be influenced by the elevation of leaf litters and root materials decomposition in summer, the surface sediments of mangrove covering field tend to acquire higher organic matter contents in summer than in winter (Zhou et al., 2010). In the winter MG sampling sites, the subsurface sediment with depth around 10–15 cm (Depth B) always showed the lowest pH value, and along the increase of depth, pH values tended to increase. In the summer MG sampling sites, pH values tended to increase along the sediment depth profile for all three layers. As for TF samples, the surface samples always showed lower pH values than subsurface samples. The redox potential and water content were generally negatively associated for TF samples, and the surface sediments always had lower redox potential than the subsurface sediments. In MG samples, the surface samples showed high water contents, especially for summer samples. Meanwhile, the redox potential values tended to decrease along the sediment depth profile in MG samples, irrespective of seasonality, which might result from the oxygen availability for the upper layers. A reverse trend for redox potential in the upper layers of MG and TF sediments was similar to that reported on the physicochemical assessment of mangrove covering field and unvegetated mudflat sediments in Yifeng Estuary, China (Zhou et al., 2010).

### Weaker seasonal dynamic of archaeal community than bacterial community

There was no significant difference between summer and winter for bacterial and archaeal 16S rRNA gene abundance (Figure 2). The most abundant five bacterial phyla remained unchanged in both seasons, for all bacterial communities (Figure 3A), except for *Cyanobacteria*, specifically enriched in surface sediments of winter (*p* < 0.001; abundance fraction mean difference between summer and winter is 26.2%). The most five abundant classes still remained the same, but with minor changes at the order level, for all archaeal communities (Figure 3B). The alpha diversity dynamics between summer and winter showed that Shannon indices of bacterial communities in winter samples were significantly lower than those in summer samples, indicating a less diverse bacterial community distribution pattern in winter (Supplementary Table S4). Chao1 index and observed species for archaeal community in winter were significantly higher than those in summer, indicating more archaeal OTUs and higher archaeal community richness in winter. Seasonality influenced the beta diversity pattern of bacterial community significantly, based on weighted UniFrac and Bray-Curtis distance matrix method but not non-weighted UniFrac distance matrix method, suggesting that both of the presence and the sequence quantity of OTUs from each phylogenetic lineage influenced the distribution pattern of bacterial communities. However, seasonality did not show any significant influence on beta diversity pattern of archaeal community. Overall, seasonality imposed more influence on the beta diversity pattern of bacterial community than archaeal community. O'sullivan et al. (2013) reported that differences of geochemical factors were small between sediment depth profiles at two different seasonal time points with average temperature difference of 8°C from Colne estuary, which indicates a weak seasonal influence on the geochemical profiles of coastal wetland sediment. Similarly, weak seasonal dynamics of microbial community in Mai Po wetland sediments could be also attributed to small geochemical profile differences.

### Sediment layer depth and seasonality on the bacterial community

Sediment depth positively influenced the bacterial community diversity as reflected by Pearson correlation analysis (Table 2). This phenomenon was also supported by one-way ANOVA analysis, suggesting that diverse indices including Shannon, Simpson and observed species indices were significantly different among four layers, and the Depth A (surface layer) acquired the lowest alpha indices (Supplementary Table S4). For the beta diversity, sediment depth and seasonality also separated microbial communities into corresponding assemblages by PCoA based on UniFrac and Bray-Curtis matrix methods (Figure 4, Supplementary Table S5). Mantel Test was applied to delineate any significant correlation between bacterial community dissimilar distance matrix and physicochemical parameter distance matrix; and results revealed that by at least one distance matrix method (UniFrac and Bray-Curtis matrix method), layer depth, water content, ${\text{NO}}_{3}^{-}$, Σ(${\text{NO}}_{3}^{-}$ + ${\text{NO}}_{2}^{-}$), redox potential and pH were influential factors, significantly correlated with bacterial community distribution pattern among all the samples (Supplementary Table S6). Furthermore, sediment depth and seasonality were the top two factors accounting for the most explained variance by MRT analysis (Figure 6). Base on the Pearson correlation analysis between the physicochemical parameters and the abundance of bacterial taxa at class level, sediment depth was also the most prevalent influential factor, significantly correlated with the class abundance, either positively or negatively (Supplementary Table S7). Bacterial 16S rRNA gene abundance was negatively correlated with sediment depth, but water contents and concentrations of ${\text{NO}}_{2}^{-}$, ${\text{NO}}_{3}^{-}$ and Σ(${\text{NO}}_{3}^{-}$ + ${\text{NO}}_{2}^{-}$) were positively correlated (Table 2). Such phenomenon on microbial abundance and community changes influenced by sediment depth has also been reported. Recently, a prokaryotic diversity study on depth profiles along the Colne estuarine gradient from brackish to marine condition revealed that prokaryotic cell numbers also decreased along the sediment depth (0–50 cm) (O'sullivan et al., 2013). It was also observed that the prokaryotic population shifted between surface and subsurface layers in two estuarine sediment depth profiles, despite of minor changes of geochemical profiles, including methane and sulfate (O'sullivan et al., 2013).

### pH as the most influential factor on shaping the archaeal community

Pearson correlation analysis between alpha diversity indices and physicochemical parameters showed that pH was positively correlated with all the diversity and richness indices, but negatively with Good's coverage. In contrast, redox potential showed a reverse trend with pH value (Table 2). ${\text{NH}}_{4}^{+}$ was positively and significantly correlated with archaeal 16S rRNA gene abundance and Shannon and Simpson indices. The organic matter content negatively influenced alpha diversity indices, including, PD tree, Chao1 and observed species, and positively influenced the Good's coverage value for all archaeal communities (Table 2). As revealed from the PCoA ordination, sediment depth also imposed significant influence on archaeal communities (Figure 4), while at the same time, archaeal communities along the sediment depth profiles within individual sampling sites also showed significant differences based on weighted UniFrac and Bray-Curtis distance matrix methods (Supplementary Table S5). Moreover, pH was also the single factor significantly correlated with the archaeal communities by both UniFrac and Bray-Curtis matrix methods based on Mantel Test (Supplementary Table S6). The MRT showed that pH was one of the most influential factors responsible for 27.57% of the explaining effect, second by layer depth with 25.74% (Figure 5B). Similar to this study, another high throughput 16S rRNA gene sequencing study on tropical soil archaeal diversity and community at regional level revealed that pH was the most important factor which partitioned archaeal community distribution at OTU level (Tripathi et al., 2013). Furthermore, non-uniform archaeal abundance response to pH alteration in an arable soil across a pH gradient (4.0–8.3) potentially reflected the variation of archaeal community composition along the change of pH (Bengtson et al., 2012).

### Stratified microbial distribution

**Figure 7:**
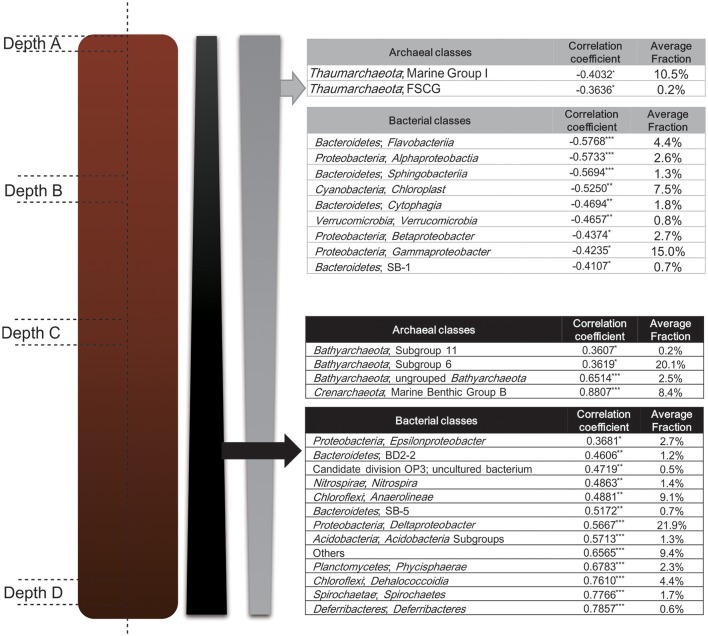
Pearson correlation analysis between sediment layer depth and abundance of groups at the class level for Bacteria and Archaea. Correlation coefficient with *p*-value score < 0.05, 0.01, 0.001 is labeled with “^*^”, “^**^”, and “^***^” respectively. Classes are ordered along the increase of correlation coefficient.

In delineating the correlation between sediment depth and taxa abundance fraction at the class level, almost all the class abundance fraction either positively or negatively correlated with the increase of sediment layer depth, except for a small portion among all the bacterial classes (ca. 6%) (Figure 7). The bacterial classes which showed significantly negative correlation with layer depth were also found to be enriched in Depth A layer (Figure 5), meanwhile, the bacterial classes which showed significantly positive correlation with layer depth were also found to be enriched in Depth B, C, D layers (Figure 5). At the same time, as for the archaeal community, Pearson correlation analysis between archaeal class abundance fraction and sediment depth also corroborated the stratified distribution of archaeal communities that revealed by LEfSe analysis (Figure 5). The major phyla and classes of bacteria and archaea discovered in Mai Po wetland were also ubiquitously found in the other natural, constructed wetlands or estuarine sediments (Wang et al., 2012; Jiang et al., 2013; Ansola et al., 2014; Arroyo et al., 2015; Lu et al., 2016).

In this study, a clear stratified distribution of most of the identified taxa at the class level was evident as the abundance fractions of most of the classes (ca. 94%) either increased or decreased along the sediment depth profile with significant statistical support. *Flavobacteriia* was mostly represented by the family *Flavobacteriaceae* in this study. Most of the members within *Flavobacteriaceae* are aerobic, capable of utilizing macromolecules, such as proteins and polysaccharides (Rosenberg et al., 2014d). The three major subordinate families of *Cytophagia, Cyclobacteriaceae, Cytophagaceae*, and *Flammeovirgaceae*, share the general physiological feature as aerobic, chemoheterotrophic and capable to degrade a variety of biomacromolecules, such as proteins and polysaccharides, and are widely distributed in aquatic and terrestrial environments (Reichenbach, 2006; Yoon et al., 2011; Rosenberg et al., 2014d). *Saprospiraceae* and WCHB1-69 mainly made up of *Sphingobacteriia* class in this study, and the former family is characterized as aerobic heterotrophs with the ability to hydrolyze complex carbon sources, and some of them could form helical gliding filaments and acquire gliding motility for predating other bacteria and algae (Rosenberg et al., 2014d). *Gammaproteobacteria* was the most abundant class, whose abundance fraction generally decreased along the depth profile in this study. While, the subordinate *Chromatiales* (mainly represented by *Ectothiorhodospiraceae*) was enriched in the Depth C layer. *Ectothiorhodospiraceae* mostly contains the phototrophic purple sulfur bacteria that conduct anoxygenic photosynthesis and some of the members could also live photoheterotrophically depending on a list of limited organic matters. Some of this family could also live purely chemoorganotrophically with oxygen or nitrate as the electron acceptors (Garrity et al., 2006). It will be reasonable that *Chromatiales* order in this study was enriched in the subsurface sediment layers, due to the strict anaerobic or facultative anaerobic property of this group of bacteria (Rosenberg et al., 2014c).

However, the other three major classes in *Gammaproteobacteria, Alteromonadales* (mainly represented by *Alteromonadaceae*), *Xanthomonadales* and *Oceanospirillales*, are generally aerobic. *Alteromonadaceae* is a group of obligately aerobic heterotrophs which prefer nutrient rich environments (Rosenberg et al., 2014c). *Xanthomonadales* is also a group of obligate aerobic chemoheterotrophs, and has a strict respiratory metabolism with oxygen as the terminal electron acceptor (Garrity et al., 2006). *Oceanospirillales* is characterized as motile with polar flagella and aerobic, and most of the members are found in marine environments (Garrity et al., 2006). *Thiotrichales* (mainly represented by *Thiotrichaceae*) and the other *Gammaproteobacteria Incertae Sedis* are lacking specific physiological description. In this study, the abundance fraction of *Gammaproteobacteria* as a whole class, showed a decreasing trend toward depth profiles, which might result from its major aerobic components (Figure 7). *Burkholderiales, Hydrogenophilales*, and *Nitrosomonadales* were the major components of *Betaproteobacteria* in this study. *Alcaligenaceae* comprised the major part of *Burkholderiales* in this study. It is characterized as aerobic, acquiring a set of strict respiratory elements with oxygen as the final terminal electron acceptor (Brenner et al., 2005). The most abundant genus within *Hydrogenophilales* was *Thiobacillus*, which characterized as facultative anaerobic and strictly chemolithotrophic; and it could oxidize reduced sulfur compounds to sulfur/sulfate in the presence of oxygen and often be found in freshwater, estuarine and marine sediments (Rosenberg et al., 2014a). *Nitrosomonadales* mainly consisted of *Nitrosomonadaceae* in this study, which is known as a group of aerobic lithoautotrophic ammonia oxidizers (Rosenberg et al., 2014a). *Rhodobacteraceae* was the major constituent part of *Alphaproteobacteria* in this study, and they are mainly characterized as aerobic photo/chemoheterotrophs (Rosenberg et al., 2014a). The uncultured *Cyanobacteria*/*Chloroplast* relatives in this study were enriched in the surface layer and drastically decreased along the depth profile, which is reasonable for their photosynthesis traits (Whitton and Potts, 2012). Only a few groups of genera within *Verrucomicrobia* are isolated so far, and most of them are found to be mesophilic carbohydrate degraders. They are widely found in marine and terrestrial habitats or vertebrate digestive tracts, and they are also reported to make up 1–10% of bacterial 16S rRNA gene composition of soils, which indicates their potential important functions in terrestrial ecology (Islam et al., 2008; Krieg et al., 2010). The major components of the classes, which showed negative correlation with sediment depth significantly, were aerobic. That could reasonably explicate their distribution preference in the upper layers.

The genera *Sulfurimonas* and *Sulfurovum* mainly comprised the class *Epsilonproteobacteria* in this study. *Sulfurimonas* is characterized as facultatively anaerobic and could grow chemolithoautotrophically depending on sulfide, sulfur, thiosulfate and hydrogen as electron donors and nitrate, nitrite and oxygen as electron acceptors (Rosenberg et al., 2014b). *Sulfurovum* is also a group of sulfur oxidizing bacteria with the similar physiology to *Sulfurimonas* (Rosenberg et al., 2014b). *Deltaproteobacteria* revealed in this study was mainly comprised of *Syntrophobacterales, Desulfobacterales, Desulfobacterales*, and *Desulfarculales*. It contains the largest anaerobic sulfate reducer groups within the above orders. Most of them could chemoorganoheterotrophically degrade and obtain energy from large organic molecules and subsequently metabolize incomplete oxidized products as acetate or complete oxidized products as carbon dioxide (Rosenberg et al., 2014b). *Deltaproteobacteria* has also been regarded as the representative benthic sediment group, and they prefer to live in sediments of fresh lake rather than water column, which is probably attributed to oxidation-reduction potential gradient between water and sediments (Tamaki et al., 2005; Ye et al., 2009). It was also reported that the *Syntrophobacterales* within *Deltaproteobacteria* and *Chromatiales* within *Gammaproteobacteria* were the most abundant bacterial order in all investigated wetland sediments under both occasional and permanent flooding conditions (Ligi et al., 2014). The *Nitrospira* and uncultured group within *Nitrospiraceae* were the major components of *Nitrospirae* in this study. *Nitrospira* is a group of chemolithoautotrophic aerobic nitrite oxidizing bacteria. While the other genera within *Nitrospiraceae* are quite physiologically diverse, for instance, *Leptospirillum*, chemolithoautotrophic aerobic and acidophilic ferrous ion oxidizers and *Thermodesulfovibrio*, anaerobic hydrogenotrophic sulfate reducers (Rosenberg et al., 2014d). The major components of *Anaerolineae* were *Anaerolineaceae* uncultured groups. This class is Gram-negative, chemoorganotrophic and could only grow under strict anaerobic conditions as described (Yamada et al., 2006). The physiological properties of those uncultured group remain unclear and need further investigation. *Dehalococcoidia* in this study was mainly comprised of *Dehalococcodiales* and currently unknown groups within the class. They are strict anaerobic and mesophilic, well-known for their anaerobic respiration on oxidizing hydrogen by halogenated organic compounds (Loeffler et al., 2013). The *Chloroflexi* phylum was reported to be abundant at deep subsurface sediments as they could occupy up to 70% of the bacterial 16S rRNA gene fraction in the 200,000-year-old Mediterranean sediment (Coolen et al., 2002). Meanwhile, tidal flat sediments from a sampling core lower than 2 m harbored 60% *Chloroflexi* sequences of all bacteria, resembling that of deep subsurface sediments (Wilms et al., 2006). *Chloroflexi* could utilize recalcitrant organic matters buried in subsurface sediments better than other microorganisms, probably explaining their high abundance in such niches (Wilms et al., 2006). Most *Phycisphaerae* within *Chloroflexi* were uncultured groups in this study, with one exception, *Phycisphaera mikurensis*, which is facultatively anaerobic and could reduce nitrate to nitrite and ferment based on D-xylose; It is fermented on a variety of sugars under aerobic conditions (Fukunaga et al., 2009; Rosenberg et al., 2014d). *Spirochaetes* was mostly represented by the type genus *Spirochaeta* in this study. *Spirochaeta* is obligate or facultative anaerobic, and chemoorganotrophic, feeding on a variety of carbohydrates as carbon and energy sources (Krieg et al., 2010). They are also found indigenous to aquatic freshwater and marine environments, such as marshes, lakes, rivers and etc (Krieg et al., 2010). The class *Deferribacteres* in this study was mostly represented by *Caldithrix*. Up to date, only two cultured *Caldithrix* species have been obtained and they are both obligate anaerobes and chemoorganoheterotrophs, capable of fermenting on di- and poly-saccharides. They could also respire anaerobically depending on hydrogen or acetate as the electron donors and nitrate as the electron acceptor (Rosenberg et al., 2014d). The other uncultured groups, including *Acidobacteria* Subgroups, *Bacteroidetes* BD2-2, SB-1 subgroups and Candidate division OP3, all lack clearly described reference on their physiological properties. Collectively, It is revealed that classes which showed positive correlation with sediment depth significantly are mainly obligate or facultative anaerobic, further implicating that it is their physiological traits toward oxygen tolerance that imposes influence on subsurface distribution preference.

The archaeal community also showed a clear stratified distribution as the aerobic groups, including *Thaumarchaeota* Marine Group I and FSCG, mainly involving with aerobic oxidization of ammonium, were enriched in the surface layer and soon decreased along depth profiles; whereas, *Bathyarchaeota* Subgroup 6, 11, ungrouped *Bathyarchaeota* and MBG-B were enriched in the lower layers and increased accordingly along depth profiles (Brochier-Armanet et al., 2012). Both *Bathyarchaeota* and MBG-B were dominant archaeal groups and distributed worldwide in anoxic marine sediments, hydrothermal vent and methane seep, and etc., (Teske and Sørensen, 2008; He et al., 2016). Besides, they were also discovered universal and dominant in coastal, intertidal sediments and estuarine sediments (Kim et al., 2005; Li et al., 2012). Recent physiological and genomic evidence suggests that both of them have an anaerobic and heterotrophic lifestyle depending on the buried organic carbons (Teske and Sørensen, 2008; Lloyd et al., 2013). The relative fraction of *Bathyarchaeota* within archaea decreased in shallow sediments in White Oak River (WOR) estuary depth profiles. Meanwhile, it was revealed that *Bathyarchaeota* Subgroup 6 preferred suboxic sediment layer with limited free sulfide (Lazar et al., 2015). The stratified archaeal community compositional patterns with *Thaumarchaeota* Marine Group I dominant at surface wetland sediments and *Bathyarchaeota* dominant at subsurface wetland sediments were also observed in the previously investigations (Webster et al., 2010; Jiang et al., 2011; Li et al., 2012). It was speculated that oxygen availability of sediment layers might be the major factor contributing to stratified distribution of archaeal community in Mai Po wetland. Oxygen level is quickly depleted below the surface sediments, and under suboxic conditions, microbial degraders will utilize organic matters using a set of terminal electron accepters with decreasing redox potential, such as ${\text{NO}}_{3}^{-}$, Mn^4+^, Fe^3+^, ${\text{SO}}_{4}^{2-}$, and ${\text{CO}}_{3}^{2-}$ (Canfield and Thamdrup, 2009; O'sullivan et al., 2013). Along the geochemical variation of depth profile, microbial community pattern will be influenced accordingly, whilst, the most influential factors would be the respiratory condition with the presence/absence of oxygen, which largely stratifies microbial community compositions (Canfield and Thamdrup, 2009; O'sullivan et al., 2013).

## Conclusion

In conclusion, the present study gives a comprehensive investigation on the bacterial and archaeal community abundance, composition and diversity in Mai Po wetland sediments, and has compared their distribution patterns in terms of depth profiles, sediment types, sampling locations and seasonality. The abundance of bacterial 16S rRNA genes in individual sampling sites shows a clear decreasing trend from surface layers to subsurface layers, irrespective of sampling sites, seasonality and sediment types. The most abundant five phyla of both bacterial and archaeal communities remain stable in terms of different sediment types. The alpha and beta diversity pattern revealed by the bacterial and archaeal community structure suggests that there is a weak seasonal dynamic of microbial community in Mai Po wetland. Meanwhile, sediment layer depth and seasonality influence the most on the bacterial community, while pH is the most influential factor on shaping the archaeal community. Stratified distribution patterns on community abundance and composition for both bacterial and archaeal communities are clear, as the aerobic groups dominate the surface layers, and anaerobic groups dominate the subsurface layers. *Thaumarchaeota* Marine Group I are dominant at surface sediments and *Bathyarchaeota* and MBG-B are dominant at subsurface. The stratified distribution pattern of microbial community revealed from this study might result from oxygen availability and gradient distribution of terminal electron accepters along the depth profile. This phenomenon has also been observed universally. More precise biogeochemical profiling, such as profiling of *in situ* physicochemical parameters and biogeochemical activity rates, on depth profiles and especially on deeper sediment layers should be conducted; and combined with advanced sequencing techniques, more insightful outcomes will be achieved on the stratified distribution of microbial communities in coastal wetlands.

## Author contributions

ZZ, JG, and ML conceived this study. ZZ and HM performed the sampling and physicochemical measurement. ZZ performed the original data analysis and drafted the original manuscript. HM, YL, JG, and ML contributed together to the final manuscript by discussing and rewriting several essential parts.

### Conflict of interest statement

The authors declare that the research was conducted in the absence of any commercial or financial relationships that could be construed as a potential conflict of interest.
