# Supplementary material for: Stratified Bacterial and Archaeal Community in Mangrove and Intertidal Wetland Mudflats Revealed by High Throughput 16S rRNA Gene Sequencing
Source: Front Microbiol. 2017 Nov 2;8:2148. doi: 10.3389/fmicb.2017.02148 (PMC5673634; doi:10.3389/fmicb.2017.02148)
Supplement: Supplementary file 1 [file Presentation1.pdf]

Supplementary Material

Stratified bacterial and archaeal community in mangrove and intertidal wetland mudflats revealed by high throughput 16S rRNA gene sequencing

Zhichao Zhou[1], Han Meng[1], Yang Liu[2], Ji-Dong Gu[1*] & Meng Li[2*]

*** Correspondence:** Ji-Dong Gu: jdgu@hku.hk; Meng Li: limeng848@szu.edu.cn

Supplementary Tables

Supplementary Table S1. Quantitative results of the abundance of bacterial and archaeal 16S rRNA gene from samples collected from Mai Po sediments

	bacterial 16S rRNA gene		archaeal 16S rRNA gene		a+b	Ratio (a/(a+b))
	average	sd	average	sd	average	(%)
MG1WinA	2.97E+10	1.60E+09	6.86E+07	5.19E+06	2.98E+10	0.23
MG1WinB	1.24E+10	7.89E+08	1.99E+08	2.88E+07	1.26E+10	1.58
MG1WinC	5.59E+09	2.58E+08	1.14E+08	1.37E+07	5.70E+09	2.00
MG1WinD	3.95E+09	8.42E+07	1.11E+08	4.44E+06	4.06E+09	2.73
MG2WinA	2.53E+10	1.27E+08	7.84E+07	5.15E+06	2.54E+10	0.31
MG2WinB	7.13E+09	3.37E+08	1.04E+08	5.25E+06	7.24E+09	1.43
MG2WinC	5.16E+09	6.84E+08	9.30E+07	6.52E+06	5.25E+09	1.77
MG2WinD	2.45E+09	3.79E+07	2.32E+08	9.71E+06	2.68E+09	8.63
MG3WinA	1.48E+10	6.02E+07	1.23E+08	8.21E+06	1.49E+10	0.82
MG3WinB	8.97E+09	4.57E+08	8.50E+07	4.13E+06	9.06E+09	0.94
MG3WinC	3.88E+09	9.92E+07	7.27E+07	7.28E+06	3.95E+09	1.84
MG3WinD	9.60E+08	3.82E+07	4.71E+07	3.39E+06	1.01E+09	4.68
TF1WinA	1.04E+10	4.22E+08	1.96E+07	6.49E+05	1.04E+10	0.19
TF1WinB	1.07E+10	6.09E+08	2.33E+08	1.37E+07	1.10E+10	2.12
TF2WinA	2.09E+10	8.47E+08	1.31E+08	8.72E+06	2.10E+10	0.62
TF2WinB	7.68E+09	1.09E+08	3.62E+08	1.41E+07	8.05E+09	4.49
TF3WinA	1.74E+10	1.06E+09	7.85E+07	4.05E+06	1.75E+10	0.45
TF3WinB	3.47E+09	1.87E+08	1.10E+08	3.06E+06	3.58E+09	3.08
MG1SumA	1.61E+10	8.57E+08	1.02E+08	9.77E+06	1.62E+10	0.63
MG1SumB	9.21E+09	3.47E+08	3.72E+08	3.18E+07	9.58E+09	3.89
MG1SumC	3.26E+09	1.39E+08	1.70E+08	1.16E+07	3.43E+09	4.96
MG2SumA	1.51E+10	7.29E+08	1.57E+08	6.10E+06	1.52E+10	1.03
MG2SumB	4.66E+09	2.09E+08	1.40E+08	4.22E+06	4.80E+09	2.92
MG2SumC	3.18E+09	2.44E+08	2.95E+08	1.19E+07	3.48E+09	8.49
MG3SumA	1.23E+10	3.65E+08	9.17E+07	6.50E+06	1.24E+10	0.74
MG3SumB	6.86E+09	4.84E+08	1.27E+08	2.94E+06	6.99E+09	1.82
MG3SumC	2.69E+09	2.52E+08	1.07E+08	4.95E+06	2.79E+09	3.83
TF1SumA	1.09E+10	6.19E+08	2.81E+08	8.58E+06	1.12E+10	2.50
TF1SumB	2.02E+09	2.21E+08	1.60E+08	2.08E+06	2.18E+09	7.35
TF2SumA	4.78E+10	1.18E+09	3.05E+08	2.25E+07	4.81E+10	0.63
TF2SumB	2.05E+09	9.04E+07	1.43E+08	6.41E+06	2.20E+09	6.50
TF3SumA	1.14E+10	1.04E+09	9.70E+07	6.46E+06	1.15E+10	0.84
TF3SumB	2.12E+09	1.12E+08	1.46E+08	8.39E+06	2.27E+09	6.45

The values are copy numbers per gram dry sediments. “sd” stands for standard deviation, “a+b” stands for archaeal and bacterial 16S rRNA gene abundance, “Ratio (a/(a+b))” stands for ratio of archaeal 16S rRNA gene abundance over archaeal and bacterial 16S rRNA gene abundance.

Supplementary Table S2. Compositional summary of all bacterial communities from 33 Mai Po wetland sediment samples at the class level

Taxon/ abundance fraction (%)	average	MG1WinA	MG1WinB	MG1WinC	MG1WinD	MG2WinA	MG2WinB	MG2WinC	MG2WinD	MG3WinA	MG3WinB	MG3WinC	MG3WinD	TF1WinA	TF1WinB	TF2WinA	TF2WinB	TF3WinA	TF3WinB	MG1SumA	MG1SumB	MG1SumC	MG2SumA	MG2SumB	MG2SumC	MG3SumA	MG3SumB	MG3SumC	TF1SumA	TF1SumB	TF2SumA	TF2SumB	TF3SumA	TF3SumB
Acidobacteria; *Acidobacteria* Subgroups	1.3	0.3	1.2	1.2	1.6	0.2	1.5	1.2	3.5	0.6	1.6	1.6	3.6	0.0	0.9	0.2	0.8	0.6	1.1	1.1	1.0	1.2	3.5	2.0	1.0	1.7	1.7	2.0	1.6	1.4	0.9	1.2	1.1	1.1
Acidobacteria; *Holophagae*	0.9	0.2	1.2	0.9	1.1	0.3	1.9	1.5	1.1	0.6	1.4	1.2	1.8	0.2	1.4	0.1	0.4	1.1	1.1	1.0	0.6	0.5	1.6	0.9	0.2	1.3	0.4	0.6	1.5	0.7	0.7	0.6	1.7	0.9
Bacteroidetes; *Bacteroidia*	0.8	0.2	2.2	1.0	0.8	1.5	1.0	1.4	0.1	0.6	2.5	0.8	0.8	0.4	0.7	0.9	0.8	0.4	0.3	1.2	0.7	0.1	0.3	0.4	0.4	0.5	0.5	0.3	0.6	0.0	2.7	0.0	0.8	0.2
Bacteroidetes; BD2-2	1.2	0.4	2.3	2.6	2.4	0.3	1.7	2.3	1.3	0.6	3.7	1.8	0.7	0.6	0.9	0.7	1.0	1.0	0.6	1.2	1.4	1.9	0.3	1.6	1.9	0.3	2.5	2.0	0.5	0.3	0.9	0.4	1.0	0.2
Bacteroidetes; *Cytophagia*	1.8	2.9	1.4	0.8	0.8	4.5	1.5	1.4	0.3	10.5	1.7	1.4	0.8	1.3	2.4	1.3	0.6	1.8	1.4	1.1	0.6	0.3	2.2	1.3	0.5	5.0	1.6	0.7	1.8	0.0	4.5	0.1	2.6	0.5
Bacteroidetes; *Flavobacteriia*	4.4	12.6	2.0	0.6	0.6	16.0	2.9	2.3	0.3	10.3	0.6	1.4	0.4	21.6	3.7	11.7	5.7	15.1	5.5	4.4	0.8	0.6	1.5	0.1	0.3	7.1	0.4	0.4	3.8	2.1	4.8	3.2	2.0	0.6
Bacteroidetes; SB-1	0.7	0.5	1.2	0.5	0.4	1.1	0.8	0.9	0.0	0.6	3.0	0.8	0.1	1.5	1.0	1.8	0.3	1.2	0.5	1.4	0.6	0.3	0.2	0.2	0.1	0.8	0.7	0.4	0.5	0.0	1.0	0.1	1.0	0.3
Bacteroidetes; SB-5	0.7	0.1	1.2	1.5	1.1	0.1	1.1	1.5	1.0	0.3	1.6	1.4	0.3	0.1	0.4	0.1	0.3	0.1	0.2	0.5	1.3	1.6	0.2	1.7	1.9	0.1	1.0	1.5	0.2	0.1	0.4	0.2	0.5	0.1
Bacteroidetes; *Sphingobacteriia*	1.3	1.7	1.2	0.9	0.5	1.4	1.5	1.5	0.1	0.8	1.1	0.6	0.1	0.9	3.2	0.9	0.9	2.1	1.2	3.2	1.1	0.2	2.3	0.2	0.2	2.7	0.6	0.2	2.1	0.2	4.9	0.3	2.5	0.6
Candidate division OP3; uncultured bacterium	0.5	0.1	0.5	0.8	1.0	0.1	0.3	0.7	0.5	0.0	0.3	1.0	0.5	0.1	0.4	0.2	0.9	0.1	1.2	0.6	0.3	2.8	0.1	0.7	1.1	0.0	0.3	0.5	0.4	0.9	0.1	0.7	0.4	0.5
Chlorobi; *Ignavibacteria*	0.6	0.1	0.6	0.4	0.7	0.2	0.8	0.7	0.6	0.3	1.0	1.0	1.3	0.3	0.5	0.2	0.3	0.3	0.3	0.7	0.4	0.2	1.8	1.2	0.3	0.5	0.7	0.5	0.8	0.1	0.6	0.1	0.8	0.2
Chloroflexi; *Anaerolineae*	9.1	1.6	8.0	12.0	13.8	1.8	7.4	9.3	9.0	3.0	9.0	8.6	9.3	1.9	8.2	2.6	9.4	5.6	7.8	8.7	11.4	13.1	8.9	12.4	12.2	5.9	13.3	13.4	7.9	16.3	6.9	18.5	11.0	11.2
Chloroflexi; *Ardenticatenia*	0.6	0.5	0.5	0.7	0.5	0.2	0.6	0.9	0.4	0.4	0.9	0.8	0.5	0.3	0.4	0.1	0.2	0.2	0.5	0.7	0.8	0.5	1.2	1.8	0.9	0.6	1.2	0.4	0.5	0.5	0.4	0.3	0.6	0.4
Chloroflexi; *Dehalococcoidia*	4.4	0.1	3.6	8.0	7.5	0.1	3.6	4.2	14.6	0.3	1.4	4.2	9.1	0.2	2.5	0.3	4.4	0.3	4.2	0.9	4.2	9.2	0.1	4.5	13.2	0.1	3.0	8.2	2.7	12.8	0.4	9.4	0.8	8.8
Cyanobacteria; *Chloroplast*	7.5	36.2	0.5	0.3	0.1	37.2	0.4	0.4	0.1	28.9	0.3	0.4	0.3	32.0	3.9	46.3	2.4	14.1	3.8	2.7	0.1	0.1	1.1	0.1	0.1	4.4	1.2	0.3	3.8	0.1	19.3	0.2	5.2	0.1
Deferribacteres; *Deferribacteres*	0.6	0.1	0.5	0.9	1.0	0.0	0.5	0.6	1.2	0.2	0.3	0.6	1.2	0.1	0.6	0.2	0.4	0.3	0.7	0.4	0.6	0.6	0.3	1.2	1.2	0.2	0.9	1.0	0.8	0.6	0.3	0.6	0.5	0.8
Gemmatimonadetes; *Gemmatimonadetes*	0.8	0.2	0.5	0.6	0.6	0.2	1.0	0.6	0.9	0.4	0.8	0.8	2.3	0.2	1.1	0.1	0.7	0.5	0.9	0.4	0.7	0.6	1.8	1.2	0.4	1.4	0.7	1.1	0.9	0.9	0.8	1.0	1.0	0.9
Lentisphaerae; R76-B128	0.5	0.9	1.7	0.4	0.4	0.3	0.4	0.8	0.1	0.8	1.1	0.9	0.1	0.1	0.4	0.5	0.2	0.1	0.4	3.5	0.7	0.1	0.9	0.1	0.1	0.6	0.3	0.2	0.5	0.0	1.4	0.0	0.2	0.1
Nitrospirae; *Nitrospira*	1.4	0.1	0.3	1.1	0.7	0.1	0.9	0.7	1.7	0.6	1.0	2.6	6.5	0.2	0.9	0.1	0.7	0.3	1.3	1.5	0.8	1.7	2.0	3.0	1.4	0.9	4.4	3.8	1.2	1.1	0.1	0.6	0.8	3.2
Planctomycetes; *Phycisphaerae*	2.3	0.2	1.7	2.8	3.7	0.2	2.2	1.9	3.4	0.7	1.0	3.1	4.1	0.3	1.6	0.5	1.7	0.6	2.6	1.4	2.8	4.3	0.7	3.2	6.5	0.6	2.6	3.6	2.0	5.1	0.7	3.2	1.6	4.4
Planctomycetes; *Planctomycetacia*	1.1	1.0	1.2	0.9	1.2	0.6	1.5	1.4	0.9	0.9	1.5	1.3	0.7	0.4	1.2	0.3	0.9	0.7	0.7	1.6	1.6	1.6	1.5	1.2	1.2	1.1	1.2	1.2	1.3	0.9	1.0	1.0	1.3	1.2
Proteobacteria; *Alphaproteobacteria*	2.6	7.3	1.9	0.8	1.0	4.9	1.7	1.3	0.7	3.0	2.3	1.9	1.8	9.8	1.6	4.1	2.3	8.5	1.9	1.5	1.4	0.6	5.0	2.4	0.4	6.9	1.8	0.6	2.7	0.3	3.3	0.6	1.8	0.6
Proteobacteria; *Betaproteobacteria*	2.7	2.1	1.4	2.9	1.6	1.8	2.2	1.8	0.6	3.4	3.7	1.7	1.8	1.2	4.2	0.7	2.2	2.0	2.3	5.5	1.5	0.9	11.4	2.4	0.5	8.2	2.5	0.8	3.6	0.8	3.9	1.9	5.3	2.1
Proteobacteria; *Deltaproteobacteria*	21.9	5.8	24.1	19.1	23.0	5.8	27.1	25.6	32.5	11.5	26.8	27.1	25.1	5.7	23.6	8.8	23.9	11.7	27.8	25.9	26.2	25.1	17.1	30.3	28.9	10.0	27.4	27.1	25.2	29.0	14.4	25.9	20.9	34.7
Proteobacteria; *Epsilonproteobacteria*	2.7	0.4	10.5	10.8	6.8	1.0	4.1	6.2	0.2	1.9	5.1	4.6	0.3	0.8	0.8	1.2	4.0	0.6	0.6	0.1	8.0	5.7	0.1	0.7	3.9	0.2	3.1	3.2	0.4	0.7	0.4	0.6	0.3	0.1
Proteobacteria; *Gammaproteobacteria*	15.0	16.5	17.2	11.8	11.0	11.7	16.7	13.4	8.8	12.2	12.2	15.0	11.6	10.8	20.5	7.0	20.8	17.7	16.1	17.2	19.6	16.0	22.4	11.8	7.5	28.3	11.4	11.0	21.0	12.0	15.5	18.8	19.2	13.2
Spirochaetae; *Spirochaetes*	1.7	0.4	1.1	2.4	3.0	1.0	1.8	1.9	3.3	0.5	0.9	1.7	2.9	0.7	1.3	1.0	2.4	0.6	2.0	1.0	1.7	1.7	0.4	2.2	2.1	0.2	2.3	2.8	1.2	2.4	0.7	1.8	2.7	2.3
Verrucomicrobia; OPB35 soil group	0.7	0.4	0.8	0.9	1.2	0.2	1.1	1.4	0.1	0.5	2.0	1.1	0.1	0.1	0.6	0.2	0.3	0.3	0.4	1.5	0.4	0.7	1.7	1.1	0.3	1.6	1.1	0.7	0.1	0.4	0.7	0.1	0.7	0.4
Verrucomicrobia; *Verrucomicrobiae*	0.8	1.3	0.5	0.2	0.1	3.2	0.6	0.3	0.0	0.7	0.2	0.1	0.0	3.4	0.6	2.2	0.4	5.7	1.2	0.3	0.1	0.1	0.2	0.1	0.0	0.3	0.1	0.1	0.1	0.2	1.8	0.2	0.8	0.1
Others	9.4	6.1	8.6	12.3	11.5	3.7	11.0	12.0	12.6	5.1	10.9	10.6	11.7	5.0	10.7	5.7	10.3	6.2	11.6	9.1	8.7	7.9	9.1	10.3	11.3	8.6	11.3	11.4	10.2	9.9	6.5	8.1	11.1	10.3

Only the taxa with average abundance fraction of all bacterial communities greater than 0.5% are listed, other bacterial taxa are all included in “Others”.

Supplementary Table S3. Compositional summary of all archaeal communities from 33 Mai Po wetland sediment samples at the class level

Taxon/ abundance fraction (%)	average	MG1WinA	MG1WinB	MG1WinC	MG1WinD	MG2WinA	MG2WinB	MG2WinC	MG2WinD	MG3WinA	MG3WinB	MG3WinC	MG3WinD	TF1WinA	TF1WinB	TF2WinA	TF2WinB	TF3WinA	TF3WinB	MG1SumA	MG1SumB	MG1SumC	MG2SumA	MG2SumB	MG2SumC	MG3SumA	MG3SumB	MG3SumC	TF1SumA	TF1SumB	TF2SumA	TF2SumB	TF3SumA	TF3SumB
Bathyarchaeota; Subgroup 1	0.4	0.8	0.6	0.5	0.3	0.4	0.4	0.4	0.2	0.2	0.3	0.4	0.3	0.6	0.5	0.5	0.6	0.4	0.7	0.2	0.4	0.5	0.0	0.3	0.4	0.1	0.4	0.4	0.4	0.9	0.4	0.8	0.5	0.9
Bathyarchaeota; Subgroup 10	0.4	0.6	0.0	0.0	0.0	0.3	0.1	0.3	0.4	0.2	0.0	0.0	0.7	0.5	0.4	0.2	0.3	0.6	1.7	0.2	0.0	0.2	0.0	0.0	0.4	0.1	0.0	0.1	0.2	1.9	0.3	0.5	0.2	1.4
Bathyarchaeota; Subgroup 11	0.2	0.3	0.0	0.0	0.0	0.1	0.2	0.3	0.5	0.0	0.1	0.1	0.7	0.5	0.1	0.1	0.1	0.3	0.2	0.1	0.0	0.2	0.0	0.1	0.5	0.0	0.0	0.1	0.1	0.7	0.1	0.3	0.1	0.2
Bathyarchaeota; Subgroup 13	0.3	0.3	0.1	0.1	0.1	0.3	0.1	0.3	0.5	0.1	0.1	0.1	0.4	0.5	0.2	0.3	0.3	0.5	0.6	0.0	0.1	0.4	0.1	0.1	0.3	0.1	0.1	0.1	0.2	1.2	0.2	0.8	0.4	1.8
Bathyarchaeota; Subgroup 15/C3	0.7	0.8	1.1	0.6	0.7	0.5	0.7	0.7	0.4	0.3	1.6	0.6	0.4	0.3	0.6	0.5	0.4	0.5	0.4	2.1	0.9	0.9	0.1	1.1	0.6	0.1	1.5	0.6	0.6	0.6	0.6	0.6	0.9	0.9
Bathyarchaeota; Subgroup 17	0.1	0.2	0.0	0.0	0.1	0.1	0.1	0.2	0.3	0.0	0.0	0.1	0.2	0.1	0.2	0.1	0.1	0.2	0.2	0.0	0.0	0.3	0.0	0.3	0.2	0.0	0.0	0.0	0.2	0.2	0.0	0.6	0.0	0.6
Bathyarchaeota; Subgroup 5b	0.2	0.2	0.2	0.1	0.1	0.1	0.1	0.3	0.2	0.1	0.1	0.2	0.2	0.6	0.2	0.2	0.2	0.4	0.6	0.1	0.0	0.2	0.0	0.1	0.2	0.0	0.1	0.2	0.2	0.7	0.1	0.5	0.1	0.6
Bathyarchaeota; Subgroup 6	20.1	15.5	21.0	26.1	22.9	12.6	13.7	12.9	15.4	12.7	31.4	38.3	32.3	22.7	14.7	13.1	9.1	17.1	16.3	35.1	17.7	20.2	8.3	26.8	27.5	4.8	26.4	29.0	12.9	25.7	19.5	18.6	23.4	20.8
Bathyarchaeota; Subgroup 8	16.8	19.0	39.6	34.8	19.3	13.8	16.5	12.6	11.7	6.7	15.0	15.4	13.9	29.7	22.5	16.2	13.6	23.0	12.9	28.8	23.4	13.5	2.4	11.1	12.8	2.1	14.3	13.2	12.4	19.6	20.6	15.4	20.3	8.3
Bathyarchaeota; ungrouped *Bathyarchaeota*	2.5	2.0	2.6	3.6	3.3	1.6	2.3	2.6	2.7	1.3	2.6	5.0	4.2	1.8	1.7	1.9	1.3	2.0	2.6	2.1	1.7	3.1	0.4	4.1	3.4	0.3	2.7	4.8	1.8	3.2	1.6	2.2	2.4	3.4
Crenarchaeota; Marine Benthic Group A	0.3	0.3	0.1	0.1	0.3	0.3	0.3	0.3	0.2	0.1	0.4	0.2	0.4	0.2	0.1	0.2	0.1	0.1	0.2	0.1	0.1	0.4	0.1	0.6	0.7	0.0	1.3	0.2	0.3	0.4	0.1	0.6	0.2	0.8
Crenarchaeota; Marine Benthic Group B	8.4	4.2	6.2	11.4	15.2	3.0	7.8	16.9	23.4	2.3	5.2	12.9	25.4	6.3	7.8	6.1	10.8	6.4	11.7	3.9	5.4	12.7	0.3	6.8	11.5	0.5	5.6	7.6	8.1	6.7	3.6	9.9	5.4	7.0
Euryarchaeota; *Halobacteria*	1.1	0.9	1.3	1.0	1.1	1.0	0.8	2.1	0.9	0.2	1.6	0.6	0.3	0.6	1.8	1.1	1.7	0.6	1.3	0.8	1.6	1.0	0.2	1.7	0.6	0.2	0.9	0.2	2.7	1.5	1.9	1.9	1.7	0.7
Euryarchaeota; *Methanococci*	0.6	0.2	0.8	0.1	0.1	0.1	0.1	0.4	0.5	0.0	0.2	0.0	0.0	0.1	0.3	0.6	1.0	0.0	0.0	1.4	2.8	0.1	0.0	0.0	0.0	0.0	0.0	0.0	0.3	0.0	11.3	0.2	0.1	0.0
Euryarchaeota; *Methanomicrobia*	2.4	4.2	4.6	0.9	1.5	1.2	0.2	0.5	4.6	0.8	0.2	0.6	1.5	1.0	6.3	2.7	5.7	4.4	0.6	2.8	16.5	4.3	0.1	0.2	2.3	0.2	0.1	0.1	0.9	1.4	3.1	2.6	1.7	0.5
Euryarchaeota; *Thermoplasmata*	34.2	35.4	20.3	20.2	34.7	27.8	49.3	46.2	37.4	10.2	25.7	22.7	18.2	32.2	41.9	53.1	54.1	40.9	48.4	14.9	28.4	41.2	5.9	39.4	38.1	2.9	45.0	43.0	57.3	35.0	30.5	44.2	32.9	51.0
Thaumarchaeota; FSCG	0.2	0.7	0.6	0.0	0.0	0.2	0.0	0.1	0.0	0.5	0.9	0.2	0.1	0.1	0.1	0.0	0.1	0.0	0.0	0.3	0.0	0.0	0.6	0.2	0.0	0.5	0.0	0.0	0.0	0.0	0.1	0.1	0.5	0.0
Thaumarchaeota; Marine Group I	10.5	13.9	0.8	0.2	0.1	36.3	6.8	2.3	0.6	63.2	13.3	2.1	0.3	1.7	0.3	2.6	0.2	2.0	1.0	6.9	0.8	0.6	79.6	6.8	0.3	87.8	0.9	0.1	1.1	0.0	5.9	0.0	8.7	0.4
Thaumarchaeota; Soil Crenarchaeotic Group(SCG)	0.2	0.2	0.1	0.0	0.0	0.2	0.4	0.2	0.0	0.8	1.0	0.2	0.2	0.1	0.1	0.0	0.0	0.1	0.0	0.1	0.0	0.0	1.8	0.2	0.0	0.4	0.0	0.0	0.1	0.0	0.1	0.0	0.3	0.0
Others	0.2	0.4	0.0	0.0	0.1	0.3	0.1	0.3	0.2	0.1	0.1	0.1	0.2	0.5	0.2	0.3	0.2	0.4	0.4	0.1	0.0	0.3	0.0	0.1	0.2	0.1	0.5	0.2	0.2	0.4	0.1	0.3	0.2	0.7

Only the taxa with average abundance fraction of all archaeal communities greater than 0.1% are listed, other archaeal taxa are all included in “Others”.

Supplementary Table S4. Alpha diversity values of microbial communities based on QIIME software

a bacteria

SampleID	Seqs/Sample	PD_whole_tree	Chao1	Good's coverage	observed species	Shannon	Simpson
MG1WinA	5380	78.211	4086.06	0.805	1537.6	8.307	0.975
MG1WinB	5380	103.25	6352.417	0.703	2282	9.995	0.996
MG1WinC	5380	110.432	5629.4	0.71	2303.9	9.974	0.996
MG1WinD	5380	106.713	6235.94	0.708	2287.3	10.018	0.996
MG2WinA	5380	70.301	3999.943	0.815	1447.7	7.737	0.951
MG2WinB	5380	116.285	7098.219	0.665	2515.5	10.263	0.997
MG2WinC	5380	112.829	6398.984	0.682	2432.3	10.094	0.995
MG2WinD	5380	92.227	4654.556	0.758	2052.9	9.774	0.993
MG3WinA	5380	91.503	5288.294	0.764	1753.6	8.298	0.969
MG3WinB	5380	100.999	5596.29	0.724	2206.1	9.994	0.997
MG3WinC	5380	120.668	6773.967	0.68	2463.8	10.274	0.997
MG3WinD	5380	98.589	4865.508	0.746	2118.2	9.952	0.996
TF1WinA	5380	69.448	3603.248	0.84	1285.9	7.535	0.957
TF1WinB	5380	116.61	5813.479	0.709	2265.6	9.954	0.996
TF2WinA	5380	71.762	3729.11	0.84	1233.6	6.94	0.924
TF2WinB	5380	114.735	5762.415	0.712	2237.5	9.88	0.996
TF3WinA	5380	91.085	4729.793	0.763	1858.3	9.257	0.994
TF3WinB	5380	127.227	6981.183	0.67	2463.6	10.031	0.993
MG1SumA	5380	107.749	5995.598	0.716	2227.5	10.026	0.997
MG1SumB	5380	98.03	5391.956	0.721	2252.6	10.048	0.997
MG1SumC	5380	104.332	5467.602	0.738	2093.2	9.76	0.995
MG2SumA	5380	107.862	4980.806	0.731	2269.1	10.256	0.998
MG2SumB	5380	114.608	5799.908	0.705	2337.7	10.142	0.997
MG2SumC	5380	104.871	5195.191	0.723	2259.8	10.093	0.997
MG3SumA	5380	107.217	5208.751	0.722	2270.4	10.088	0.997
MG3SumB	5380	104.327	5300.632	0.735	2153.7	9.944	0.997
MG3SumC	5380	100.171	5581.211	0.727	2190.5	9.967	0.996
TF1SumA	5380	115.235	7034.494	0.672	2464.7	10.203	0.997
TF1SumB	5380	93.88	4992.111	0.752	2015.6	9.577	0.993
TF2SumA	5380	97.512	5652.426	0.745	1952.2	9.204	0.987
TF2SumB	5380	92.231	4804.756	0.753	1954.6	9.327	0.992
TF3SumA	5380	119.922	7367.764	0.661	2541.7	10.311	0.997
TF3SumB	5380	109.356	5719.668	0.706	2269	9.628	0.986

b archaea

SampleID	Seqs/Sample	PD_whole_tree	Chao1	Good's coverage	observed species	Shannon	Simpson
MG1WinA	9270	38.779	2087.766	0.943	942.9	7.224	0.982
MG1WinB	9270	34.47	1636.872	0.951	813.9	6.394	0.958
MG1WinC	9270	31.306	1500.96	0.955	761.9	6.196	0.958
MG1WinD	9270	33.911	1620.928	0.953	799	6.426	0.961
MG2WinA	9270	38.166	2327.33	0.939	923	6.532	0.962
MG2WinB	9270	42.141	2657.748	0.925	1108.7	7.027	0.978
MG2WinC	9270	50.263	2730.396	0.923	1220.9	7.653	0.986
MG2WinD	9270	42.966	2561.607	0.929	1103	7.169	0.977
MG3WinA	9270	30.31	1705.75	0.953	736	5.22	0.863
MG3WinB	9270	32.529	1548.821	0.955	756.9	6.561	0.974
MG3WinC	9270	34.705	2109.804	0.946	834	6.555	0.969
MG3WinD	9270	33.29	1867.95	0.947	865.9	6.939	0.978
TF1WinA	9270	42.827	2642.952	0.924	1193	7.289	0.978
TF1WinB	9270	46.15	2464.391	0.926	1180	7.328	0.98
TF2WinA	9270	45.996	2373.281	0.927	1157.9	7.3	0.978
TF2WinB	9270	49.051	3167.501	0.913	1283.8	7.478	0.982
TF3WinA	9270	47.645	3194.793	0.911	1321.9	7.447	0.979
TF3WinB	9270	54.336	3475.325	0.903	1460.9	7.804	0.983
MG1SumA	9270	26.974	1000.25	0.971	563	6.272	0.97
MG1SumB	9270	33.112	1513.883	0.957	743	6.645	0.972
MG1SumC	9270	37.275	1852.655	0.947	850	6.713	0.973
MG2SumA	9270	14.859	638.394	0.982	317	4.062	0.853
MG2SumB	9270	37.629	2069.118	0.943	921.9	6.912	0.977
MG2SumC	9270	36.85	2051.402	0.942	953.8	7.125	0.98
MG3SumA	9270	14.519	618.442	0.981	329	3.285	0.744
MG3SumB	9270	29.214	1652.72	0.957	684.9	5.282	0.888
MG3SumC	9270	28.285	1521.115	0.954	735	5.572	0.909
TF1SumA	9270	43.716	2530.864	0.929	1123	7.33	0.981
TF1SumB	9270	39.591	2185.632	0.938	986.8	7.216	0.982
TF2SumA	9270	43.696	2268.885	0.935	1021.8	6.928	0.971
TF2SumB	9270	44.299	2442.131	0.931	1077	7.388	0.984
TF3SumA	9270	43.913	2259.683	0.935	1037.9	7.204	0.98
TF3SumB	9270	46.163	2604.044	0.925	1180.9	7.528	0.983

c microorganism

SampleID	Seqs/Sample	PD_whole_tree	Chao1	Good's coverage	observed species	Shannon	Simpson
MG1WinA	6930	94.501	4140.369	0.831	1804.7	8.376	0.974
MG1WinB	6930	137.876	7547.42	0.714	2883.7	10.312	0.997
MG1WinC	6930	141.493	7243.655	0.717	2876.3	10.28	0.997
MG1WinD	6930	139.733	7265.176	0.721	2848.3	10.321	0.997
MG2WinA	6930	97.708	5049.217	0.813	1867.6	8.037	0.955
MG2WinB	6930	151.8	9013.928	0.676	3135.8	10.508	0.997
MG2WinC	6930	159.028	8419.702	0.683	3141.6	10.522	0.996
MG2WinD	6930	117.664	6120.428	0.764	2560.3	10.097	0.996
MG3WinA	6930	119.466	5984.861	0.778	2176.3	8.448	0.968
MG3WinB	6930	140.897	7133.837	0.725	2825.1	10.368	0.998
MG3WinC	6930	153.652	8183.859	0.694	3053.9	10.551	0.998
MG3WinD	6930	120.043	5850.58	0.774	2489.4	10.128	0.997
TF1WinA	6930	98.022	4478.264	0.834	1717.2	7.883	0.961
TF1WinB	6930	149.308	7451.82	0.712	2909.2	10.358	0.997
TF2WinA	6930	98.782	4872.215	0.831	1681.6	7.486	0.939
TF2WinB	6930	147.753	8075.027	0.717	2813.8	10.25	0.997
TF3WinA	6930	115.15	6837.659	0.762	2346.8	9.464	0.994
TF3WinB	6930	155.831	9176.429	0.685	3038.3	10.351	0.996
MG1SumA	6930	140.45	7404.43	0.723	2833.4	10.369	0.998
MG1SumB	6930	131.794	6628.815	0.732	2820.5	10.327	0.997
MG1SumC	6930	127.945	6654.357	0.752	2556.3	9.992	0.996
MG2SumA	6930	124.735	5383.748	0.764	2629	10.185	0.997
MG2SumB	6930	148.013	7307.568	0.72	2925	10.526	0.998
MG2SumC	6930	131.481	6567.604	0.744	2692.6	10.212	0.997
MG3SumA	6930	127.241	5732.945	0.752	2650.1	10.09	0.996
MG3SumB	6930	132.878	6267.192	0.742	2734.1	10.262	0.997
MG3SumC	6930	123.225	6564.033	0.752	2589.2	9.96	0.995
TF1SumA	6930	150.801	8537.877	0.681	3133	10.532	0.997
TF1SumB	6930	121.62	6170.686	0.765	2498.3	9.953	0.995
TF2SumA	6930	130.104	6815.248	0.747	2528.2	9.543	0.987
TF2SumB	6930	123.96	6295.866	0.759	2500.9	9.864	0.995
TF3SumA	6930	165.124	8520.091	0.68	3183.8	10.622	0.998
TF3SumB	6930	143.503	7499.473	0.719	2790.2	9.949	0.991

d bacteria

SampleID	Seqs/Sample	PD_whole_tree	Chao1	Good's coverage	observed species	Shannon	Simpson
Depth A average	5380	*93.98*	5139.7	*0.756*	*1903.5*	*9.014*	*0.979*
Depth A SD	0	18.13	1225.8	0.060	456.7	1.208	0.024
Depth A CV	0.00%	19.29%	23.85%	7.94%	23.99%	13.40%	2.46%
Depth B average	5380	*107.63*	5801.1	*0.713*	*2246.1*	*9.899*	*0.995*
Depth B SD	0.000	10.59	706.7	0.027	159.2	0.263	0.003
Depth B CV	0.00%	9.84%	12.18%	3.82%	7.09%	2.65%	0.33%
Depth C average	5380	*108.88*	5841.1	*0.710*	*2290.6*	*10.027*	*0.996*
Depth C SD	0	7.34	608.4	0.024	141.6	0.172	0.001
Depth C CV	0.00%	6.74%	10.42%	3.41%	6.18%	1.71%	0.09%
Depth D average	5380	*99.18*	5252.0	*0.737*	*2152.8*	*9.915*	*0.995*
Depth D SD	0	7.26	858.6	0.026	121.0	0.126	0.002
Depth D CV	0.00%	7.32%	16.35%	3.54%	5.62%	1.27%	0.17%
Winter group average	*5380*	*99.60*	*5422.2*	*0.739*	*2041.4*	*9.349**	*0.984*
Winter group SD	0	18.08	1118.6	0.056	419.3	1.070	0.021
Winter group CV	0.00%	18.15%	20.63%	7.64%	20.54%	11.45%	2.13%
Summer group average	*5380*	*105.15*	*5632.9*	*0.720*	*2216.8*	*9.905**	*0.995*
Summer group SD	0	7.90	718.8	0.026	167.4	0.335	0.004
Summer group CV	0.00%	7.52%	12.76%	3.65%	7.55%	3.38%	0.38%
Mangrove covering field average	*5380*	*102.44*	5519.1	0.728	2164.5	9.762	0.992
Mangrove covering field SD	0	11.92	791.4	0.037	274.2	0.710	0.012
Mangrove covering field CV	0.00%	11.64%	14.34%	5.02%	12.67%	7.27%	1.21%
Intertidal mudflats average	*5380*	*101.58*	5515.9	0.735	2045.2	9.321	0.984
Intertidal mudflats SD	0	18.67	1218.8	0.060	428.6	1.045	0.022
Intertidal mudflats CV	0.00%	18.38%	22.10%	8.13%	20.96%	11.21%	2.23%
Site 1 group average	5380	100.35	5509.3	0.734	2092.4	9.582	0.990
Site 1 group SD	0	14.83	992.5	0.049	359.9	0.854	0.013
Site 1 group CV	0.00%	14.78%	18.01%	6.65%	17.20%	8.92%	1.29%
Site 2 group average	5380	99.57	5279.7	0.739	2063.0	9.428	0.984
Site 2 group SD	0	16.58	997.9	0.052	402.4	1.105	0.024
Site 2 group CV	0.00%	16.66%	18.90%	7.10%	19.50%	11.72%	2.45%
Site 3 group average	5380	106.46	5764.8	0.718	2208.1	9.795	0.993
Site 3 group SD	0	11.91	881.1	0.035	242.9	0.575	0.009
Site 3 group CV	0.00%	11.19%	15.28%	4.92%	11.00%	5.88%	0.86%

e archaea

SampleID	Seqs/Sample	PD_whole_tree	Chao1	Good's coverage	observed species	Shannon	Simpson
Depth A average	9270	35.95	1971	0.944	888.9	6.341	*0.937*
Depth A SD	0	11.67	817.7	0.023	333.2	1.405	0.076
Depth A CV	0.00%	32.46%	41.49%	2.43%	37.48%	22.16%	8.12%
Depth B average	9270	40.72	2285	0.935	1017.0	6.964	*0.970*
Depth B SD	0	7.60	640.0	0.018	240.8	0.680	0.027
Depth B CV	0.00%	18.67%	28.01%	1.91%	23.68%	9.77%	2.77%
Depth C average	9270	36.45	1961	0.945	892.6	6.636	*0.963*
Depth C SD	0	7.59	455.9	0.012	178.2	0.723	0.028
Depth C CV	0.00%	20.81%	23.25%	1.23%	19.96%	10.89%	2.90%
Depth D average	9270	36.72	2017	0.943	922.6	6.845	*0.972*
Depth D SD	0	5.42	487.7	0.012	159.7	0.380	0.010
Depth D CV	0.00%	14.75%	24.18%	1.32%	17.31%	5.56%	0.98%
Winter group average	9270.000	40.49	2315*	0.935*	1026.0*	*6.919*	*0.968*
Winter group SD	0.000	7.33	603.3	0.017	225.7	0.636	0.028
Winter group CV	0.00%	18.10%	26.06%	1.78%	22.00%	9.19%	2.86%
Summer group average	9270.000	34.67	1814*	0.949*	835.0*	*6.364*	*0.943*
Summer group SD	0.000	10.17	647.8	0.018	270.1	1.275	0.068
Summer group CV	0.00%	29.33%	35.71%	1.92%	32.35%	20.03%	7.24%
Mangrove covering field average	9270	33.41***	1774.9***	0.950***	807.8***	*6.274****	*0.943**
Mangrove covering field SD	0	8.22	572.5	0.015	221.9	1.067	0.060
Mangrove covering field CV	0.00%	24.61%	32.25%	1.62%	27.47%	17.00%	6.40%
Intertidal mudflats average	9270	45.62***	2634.1***	0.925***	1168.7***	*7.353****	*0.980**
Intertidal mudflats SD	0	3.68	418.5	0.011	136.6	0.211	0.003
Intertidal mudflats CV	0.00%	8.06%	15.89%	1.15%	11.69%	2.87%	0.35%
Site 1 group average	9270	37.10	1912.5	0.945	905.1	6.821	*0.972*
Site 1 group SD	0	5.80	514.9	0.015	200.1	0.461	0.009
Site 1 group CV	0.00%	15.64%	26.92%	1.55%	22.11%	6.76%	0.97%
Site 2 group average	9270	40.54	2298.9	0.935	1008.1	6.870	*0.966*
Site 2 group SD	0	9.57	635.4	0.018	257.5	0.981	0.038
Site 2 group CV	0.00%	23.62%	27.64%	1.91%	25.54%	14.28%	3.94%
Site 3 group average	9270	35.90	2050.8	0.942	903.9	6.309	*0.932*
Site 3 group SD	0	11.22	811.6	0.022	322.9	1.352	0.076
Site 3 group CV	0.00%	31.24%	39.57%	2.38%	35.72%	21.42%	8.14%

f microorganism

SampleID	Seqs/Sample	PD_whole_tree	Chao1	Good's coverage	observed species	Shannon	Simpson
Depth A average	6930	121.84	6146.4	0.766	2379.3	9.253	0.980
Depth A SD	0	22.65	1485.5	0.054	534.2	1.142	0.020
Depth A CV	0.00%	18.59%	24.17%	7.09%	22.45%	12.34%	2.08%
Depth B average	6930	140.44	7380.7	0.722	2822.9	10.252	0.996
Depth B SD	0	10.99	997.0	0.026	186.8	0.217	0.002
Depth B CV	0.00%	7.83%	13.51%	3.59%	6.62%	2.11%	0.19%
Depth C average	6930	139.47	7272.2	0.724	2818.3	10.253	0.997
Depth C SD	0	14.48	840.0	0.030	245.1	0.252	0.001
Depth C CV	0.00%	10.38%	11.55%	4.19%	8.70%	2.46%	0.11%
Depth D average	6930	125.81	6412.1	0.753	2632.7	10.182	0.997
Depth D SD	0	12.11	751.0	0.028	190.1	0.121	0.001
Depth D CV	0.00%	9.63%	11.71%	3.74%	7.22%	1.19%	0.06%
Winter group average	6930	129.93	6824.7	0.746	2565.0	9.652	0.986*
Winter group SD	0	22.39	1524.0	0.054	509.7	1.068	0.018
Winter group CV	0.00%	17.23%	22.33%	7.22%	19.87%	11.07%	1.87%
Summer group average	6930	134.86	6823.3	0.735	2737.6	10.159	0.996*
Summer group SD	0	12.35	899.3	0.027	214.3	0.293	0.003
Summer group CV	0.00%	9.16%	13.18%	3.64%	7.83%	2.88%	0.30%
Mangrove covering field average	6930	131.51	6688.7	0.741	2671.1	9.994	0.992
Mangrove covering field SD	0	16.49	1154.2	0.039	357.2	0.737	0.012
Mangrove covering field CV	0.00%	12.54%	17.26%	5.20%	13.37%	7.37%	1.17%
Intertidal mudflats average	6930	133.33	7060.9	0.741	2595.1	9.688	0.987
Intertidal mudflats SD	0	22.15	1449.4	0.053	496.0	1.006	0.018
Intertidal mudflats CV	0.00%	16.61%	20.53%	7.10%	19.11%	10.39%	1.85%
Site 1 group average	6930	130.32	6683.9	0.744	2625.5	9.882	0.991
Site 1 group SD	0	18.88	1324.6	0.049	460.2	0.889	0.012
Site 1 group CV	0.00%	14.48%	19.82%	6.57%	17.53%	9.00%	1.23%
Site 2 group average	6930	130.09	6720.1	0.747	2588.8	9.748	0.987
Site 2 group SD	0	20.48	1374.6	0.048	462.2	1.033	0.020
Site 2 group CV	0.00%	15.74%	20.46%	6.41%	17.85%	10.59%	2.05%
Site 3 group average	6930	136.09	7068.3	0.733	2716.1	10.018	0.993
Site 3 group SD	0	16.87	1156.5	0.035	308.9	0.611	0.009
Site 3 group CV	0.00%	12.40%	16.36%	4.77%	11.37%	6.10%	0.88%

a) bacterial community; b) archaeal community; c) microbial community; d) summary of bacteria diversity values in four categories; e) summary of archaea diversity values in four categories; f) summary of microorganism diversity values in four categories. SD stands for standard deviation; CV stands for coefficient of variation.

"*", "**", "***" mean significantly different level between groups within categories as "< 0.05", "< 0.01", "< 0.001". Blue labeled average values mean there are significant intergroup difference among average values of four groups within "Depth" category. Significant test of categories with two groups, such as seasons and sediment types, is

conducted by unpaired *t* test. Significant test of categories with above two groups, such as sediment depth and sampling sites, is conducted by one way ANOVA with Dunnett T3 *post hoc* test. The rejected groups by homogeneity of variance test (shown by average values) are in italic.

Supplementary Table S5. Statistical significance test based on unweighted, weighted UniFrac and Bray-Curtis distance matrices, which obtained from beta diversity analysis in term of four categories

			anosim		adonis	
	Categories	Distance matrix	*R* statistic	*p*-value	R^2	*p*-value
bacteria	Depth	unweighted	0.4238***	0.001***	0.1758***	0.001***
		weighted	0.3237***	0.001***	0.4425***	0.001***
		Bray-Curtis	0.3944***	0.001***	0.2402***	0.001***
	Seasons	unweighted	0.0469	0.144	0.0376	0.099
		weighted	0.0729	0.060	0.1059*	0.031*
		Bray-Curtis	0.0873*	0.046*	0.0538*	0.028*
	Sediment Types	unweighted	0.2471**	0.008**	0.0596**	0.003**
		weighted	0.1389*	0.045*	0.0658	0.092
		Bray-Curtis	0.2576**	0.006**	0.0978***	0.001***
	Sites	unweighted	-0.0337	0.762	0.0314	0.363
		weighted	-0.0491	0.957	0.0141	0.758
		Bray-Curtis	-0.0332	0.751	0.0302	0.449
archaea	Depth	unweighted	0.1108	0.053	0.1275**	0.006**
		weighted	0.1324*	0.032*	0.2762*	0.013*
		Bray-Curtis	0.1823**	0.008**	0.2106**	0.002**
	Seasons	unweighted	0.0178	0.278	0.0318	0.369
		weighted	-0.0042	0.457	0.0156	0.599
		Bray-Curtis	0.0080	0.341	0.025	0.598
	Sediment Types	unweighted	0.2862***	0.001***	0.0844***	0.001***
		weighted	0.0552	0.197	0.1427**	0.009**
		Bray-Curtis	0.2107**	0.008**	0.1438***	0.001***
	Sites	unweighted	0.0902*	0.040*	0.0407	0.060
		weighted	0.0242	0.223	0.0360	0.292
		Bray-Curtis	0.0959*	0.021*	0.0608*	0.045*
microorganisms	Depth	unweighted	0.4514***	0.001***	0.1784***	0.001***
		weighted	0.4262***	0.001***	0.5363***	0.001***
		Bray-Curtis	0.4100***	0.001***	0.2467***	0.001***
	Seasons	unweighted	0.0251	0.218	0.0351	0.178
		weighted	0.0725	0.084	0.0958*	0.047*
		Bray-Curtis	0.0769	0.069	0.0504	0.059
	Sediment Types	unweighted	0.2515**	0.005**	0.0604**	0.003**
		weighted	0.1121	0.065	0.0481	0.181
		Bray-Curtis	0.2318**	0.005**	0.0935***	0.001***
	Sites	unweighted	-0.0181	0.576	0.0323	0.313
		weighted	-0.0612	0.974	0.0103	0.821
		Bray-Curtis	-0.0299	0.723	0.0304	0.418

Scores with *p*-value less than 0.05 are labeled with one star, scores with *p*-value less than 0.01 are labeled with two stars, scores with *p*-value less than 0.001 were labeled are with three stars.

Supplementary Table S6. Mantel Test based on the beta diversity results by unweighted, weighted UniFrac matrix and Bray-Curtis (non-phylogenetic) matrix measurement

	DM1	DM2	Number of entries	Mantel r statistic	*p*-value
bacteria	unweighted_unifrac_dm	Depth	33	**0.3680**	**0.001**
	unweighted_unifrac_dm	pH	33	**0.1507**	**0.030**
	unweighted_unifrac_dm	Redox	33	**0.1661**	**0.028**
	unweighted_unifrac_dm	Water content	33	**0.2220**	**0.009**
	unweighted_unifrac_dm	Ammonium	33	0.0417	0.500
	unweighted_unifrac_dm	Nitrite	33	0.1274	0.110
	unweighted_unifrac_dm	Nitrate	33	**0.2621**	**0.007**
	unweighted_unifrac_dm	Nitrite+Nitrate	33	**0.2616**	**0.010**
	unweighted_unifrac_dm	Organic matters	33	-0.0091	0.924
	weighted_unifrac_dm	Depth	33	**0.2853**	**0.012**
	weighted_unifrac_dm	pH	33	-0.0237	0.779
	weighted_unifrac_dm	Redox	33	0.1807	0.051
	weighted_unifrac_dm	Water content	33	**0.3366**	**0.009**
	weighted_unifrac_dm	Ammonium	33	-0.0217	0.772
	weighted_unifrac_dm	Nitrite	33	0.2137	0.062
	weighted_unifrac_dm	Nitrate	33	**0.3134**	**0.014**
	weighted_unifrac_dm	Nitrite+Nitrate	33	**0.3163**	**0.026**
	weighted_unifrac_dm	Organic matters	33	-0.0897	0.375
	bray_curtis_dm	Depth	33	**0.3260**	**0.001**
	bray_curtis_dm	pH	33	0.1257	0.065
	bray_curtis_dm	Redox	33	**0.2131**	**0.009**
	bray_curtis_dm	Water content	33	**0.2815**	**0.003**
	bray_curtis_dm	Ammonium	33	0.0441	0.510
	bray_curtis_dm	Nitrite	33	0.1579	0.054
	bray_curtis_dm	Nitrate	33	**0.2509**	**0.002**
	bray_curtis_dm	Nitrite+Nitrate	33	**0.2514**	**0.003**
	bray_curtis_dm	Organic matters	33	0.0342	0.716

	DM1	DM2	Number of entries	Mantel r statistic	*p*-value
archaea	unweighted_unifrac_dm	Depth	33	0.0986	0.290
	unweighted_unifrac_dm	pH	33	**0.3275**	**0.007**
	unweighted_unifrac_dm	Redox	33	**0.2198**	**0.033**
	unweighted_unifrac_dm	Water content	33	0.0600	0.605
	unweighted_unifrac_dm	Ammonium	33	-0.0035	0.968
	unweighted_unifrac_dm	Nitrite	33	-0.0626	0.502
	unweighted_unifrac_dm	Nitrate	33	0.0593	0.482
	unweighted_unifrac_dm	Nitrite+Nitrate	33	0.0466	0.641
	unweighted_unifrac_dm	Organic matters	33	0.0518	0.567
	weighted_unifrac_dm	Depth	33	0.1217	0.250
	weighted_unifrac_dm	pH	33	**0.3815**	**0.003**
	weighted_unifrac_dm	Redox	33	0.1458	0.151
	weighted_unifrac_dm	Water content	33	-0.0696	0.647
	weighted_unifrac_dm	Ammonium	33	-0.0491	0.663
	weighted_unifrac_dm	Nitrite	33	-0.0646	0.587
	weighted_unifrac_dm	Nitrate	33	0.0196	0.881
	weighted_unifrac_dm	Nitrite+Nitrate	33	0.0059	0.972
	weighted_unifrac_dm	Organic matters	33	-0.0558	0.571
	bray_curtis_dm	Depth	33	0.1600	0.088
	bray_curtis_dm	pH	33	**0.3277**	**0.007**
	bray_curtis_dm	Redox	33	0.1384	0.137
	bray_curtis_dm	Water content	33	-0.0108	0.930
	bray_curtis_dm	Ammonium	33	-0.0071	0.943
	bray_curtis_dm	Nitrite	33	-0.0746	0.464
	bray_curtis_dm	Nitrate	33	-0.0131	0.896
	bray_curtis_dm	Nitrite+Nitrate	33	-0.0248	0.822
	bray_curtis_dm	Organic matters	33	0.1153	0.215

The physicochemical parameter distance matrices are generated by QIIME software except for two samples with missing values. The significant scores (p-value < 0.05) are highlighted.

Supplementary Table S7. Pearson correlation analysis between physicochemical parameters and abundance fractions of groups at class level of a) bacterial and b) archaeal communities

	Depth	pH	Redox	Water content	Ammonium	Nitrite	Nitrate	Nitrate+ Nitrite	Organic matters
**Taxon proportion (Bacteria)**									
Acidobacteria; ungrouped *Acidobacteria*	0.3889*	-0.3965*	0.4438**	-0.5165**	-0.2170	-0.3630*	-0.1772	-0.1971	0.1982
Acidobacteria; *Holophagae*	0.2086	-0.3564*	0.1575	-0.2375	-0.0389	-0.2930	-0.2706	-0.2826	-0.1120
Acidobacteria; Subgroup 22	0.6096***	-0.0344	0.1241	-0.4801**	-0.0805	-0.3541*	-0.2647	-0.2812	-0.2355
Bacteroidetes; BD2-2	0.4606**	-0.2199	0.2022	-0.1986	-0.1396	-0.3180	-0.3505*	-0.3620*	0.5138**
Bacteroidetes; *Bacteroidia*	-0.1286	-0.3493*	-0.0371	0.2867	-0.1095	0.2325	-0.0515	-0.0335	0.2483
Bacteroidetes; *Cytophagia*	-0.4694**	-0.3982*	0.1324	0.0870	-0.3231	0.0876	0.2745	0.2721	-0.0294
Bacteroidetes; *Flavobacteriia*	-0.5768***	-0.0716	-0.1893	0.4327*	-0.2956	0.3525*	0.3648*	0.3781*	-0.1794
Bacteroidetes; SB-1	-0.4107*	-0.3584*	-0.0903	0.3644*	-0.1628	0.1833	-0.0129	0.0004	0.1211
Bacteroidetes; SB-5	0.5172**	-0.1875	0.2617	-0.3720*	-0.0865	-0.3912*	-0.3276	-0.3449*	0.4829**
Bacteroidetes; *Sphingobacteriia*	-0.5694***	-0.2337	-0.1032	0.4918**	-0.0028	0.5475***	0.3571*	0.3845*	-0.0568
Candidate division OP3; uncultured bacterium	0.4719**	0.4138*	-0.1420	-0.2536	0.4331*	-0.2195	-0.3140	-0.3197	0.0299
Chlorobi; *Ignavibacteria*	0.1732	-0.5756***	0.3605*	-0.3207	-0.3321	-0.1964	-0.1223	-0.1322	0.0732
Chloroflexi; *Anaerolineae*	0.4881**	0.4294*	0.0008	-0.4332*	0.4653**	-0.2563	-0.4102*	-0.4154*	0.0975
Chloroflexi; *Ardenticatenia*	0.0772	-0.4206*	0.5294**	-0.3547*	-0.2073	-0.2757	-0.0601	-0.0775	0.3823*
Chloroflexi; *Dehalococcoidia*	0.7610***	0.4391*	-0.0042	-0.4310*	0.2981	-0.3443*	-0.3678*	-0.3805*	-0.0142
Cyanobacteria; *Chloroplast*	-0.5250**	-0.0444	-0.2195	0.5403**	-0.3367	0.3877*	0.4782**	0.4904*	-0.0831
Deferribacteres; *Deferribacteres*	0.7857***	0.2510	0.1195	-0.4851**	0.0528	-0.3105	-0.3945*	-0.4039*	0.1401
Gemmatimonadetes; *Gemmatimonadetes*	0.2981	-0.0630	0.1325	-0.5187**	0.0765	-0.2135	-0.2016	-0.2102	-0.1985
Lentisphaerae; R76-B128	-0.2917	-0.4477**	0.3439	0.3259	-0.0610	0.0958	0.1673	0.1688	0.2827
Nitrospirae; *Nitrospira*	0.4863**	0.0697	0.2607	-0.6178***	-0.0717	-0.2752	-0.2262	-0.2384	-0.0418
Planctomycetes; *Phycisphaerae*	0.6783***	0.4057*	0.0345	-0.4608**	0.3200	-0.3222	-0.3818*	-0.3925*	-0.0125
Planctomycetes; *Planctomycetacia*	0.0923	-0.2444	0.3764*	-0.2375	0.2472	-0.2617	-0.0534	-0.0701	0.1452
Proteobacteria; *Alphaproteobacteria*	-0.5733***	-0.3201	-0.0520	0.3817*	-0.4342*	0.3381	0.4893**	0.4977**	-0.1023
Proteobacteria; *Betaproteobacteria*	-0.4374*	-0.5005**	0.3102	0.0129	-0.1268	0.0455	0.1774	0.1752	0.0683
Proteobacteria; *Gammaproteobacteria*	-0.4235*	-0.1034	0.0092	0.0919	0.2780	0.0938	0.1946	0.1951	-0.1516
Proteobacteria; *Deltaproteobacteria*	0.5667***	0.2073	0.0958	-0.4861**	0.3972*	-0.3341	-0.5034**	-0.5111**	-0.0811
Proteobacteria; *Epsilonproteobacteria*	0.3681*	-0.0843	0.1447	-0.0807	0.0899	-0.2969	-0.3201	-0.3310	0.5925***
Spirochaetae; *Spirochaetes*	0.7766***	0.4914**	-0.0348	-0.4353*	0.1872	-0.2684	-0.4465**	-0.4514**	0.0179
Verrucomicrobia; OPB35 soil group	-0.0285	-0.5771***	0.4968**	-0.1613	-0.1881	-0.2984	-0.0870	-0.1052	0.3860*
Verrucomicrobia; *Verrucomicrobiae*	-0.4657**	-0.0289	-0.4138*	0.4603**	-0.2773	0.3407	0.1693	0.1880	-0.1606
Others	0.6565***	0.0561	0.2061	-0.4245*	0.1181	-0.3678*	-0.4031*	-0.4163*	0.1180

	Depth	pH	Redox	Water content	Ammonium	Nitrite	Nitrate	Nitrate+ Nitrite	Organic matters
**Taxon proportion (Archaea)**									
Crenarchaeota; Marine Benthic Group A	0.1923	0.2711	0.0745	-0.2723	0.0346	-0.0611	-0.0749	-0.0769	-0.0821
Crenarchaeota; Marine Benthic Group B	0.8807***	0.3195	-0.1186	-0.2914	0.0833	-0.3028	-0.3717*	-0.3814*	-0.0956
Bathyarchaeota; Subgroup 1	-0.0411	0.6319***	-0.3504*	0.2424	0.5500***	0.2469	0.1866	0.1980	-0.2724
Bathyarchaeota; Subgroup 10	0.0216	0.4611**	-0.2472	0.0430	0.4032*	0.1047	0.0224	0.0291	-0.5045**
Bathyarchaeota; Subgroup 11	0.3607*	0.2862	-0.1130	-0.1430	0.0809	-0.0721	-0.0332	-0.0371	-0.3865*
Bathyarchaeota; Subgroup 13	0.0392	0.5379**	-0.3144	0.0014	0.4793**	0.1160	-0.0529	-0.0431	-0.4819**
Bathyarchaeota; Subgroup 15/C3	-0.0297	-0.0926	0.2824	0.0971	0.0930	-0.0471	-0.0238	-0.0264	0.3354
Bathyarchaeota; Subgroup 17	0.2589	0.5099**	-0.3008	-0.0863	0.4471**	-0.0894	-0.1037	-0.1067	-0.4046*
Bathyarchaeota; Subgroup 5b	0.0075	0.4558**	-0.3335	0.0118	0.3697*	0.1314	-0.1167	-0.1038	-0.4603**
Bathyarchaeota; Subgroup 6	0.3619*	0.0418	0.1856	-0.2471	0.0587	-0.1105	-0.1797	-0.1818	0.2606
Bathyarchaeota; Subgroup 8	-0.0240	0.0931	-0.0233	0.3468*	0.1417	0.2540	-0.0046	0.0133	0.4039*
Bathyarchaeota; ungrouped *Bathyarchaeota*	0.6514***	0.2292	0.0674	-0.4169*	0.0449	-0.3058	-0.2806	-0.2933	0.1559
Euryarchaeota; *Halobacteria*	-0.1213	0.3482*	-0.4200*	0.3108	0.4362*	0.3347	-0.0076	0.0161	-0.1110
Euryarchaeota; *Methanococci*	-0.1837	0.1015	-0.3583*	0.3642*	0.1869	0.6910***	0.2155	0.2574	-0.0139
Euryarchaeota; *Methanomicrobia*	0.0157	0.3092	-0.2170	0.2392	0.4486**	0.1107	0.0344	0.0410	-0.0122
Euryarchaeota; *Thermoplasmata*	0.0821	0.5564***	-0.5428**	0.1683	0.2284	0.2154	-0.0881	-0.0703	-0.3651*
Thaumarchaeota; FSCG	-0.3636*	-0.6324***	0.3835*	0.1501	-0.2399	-0.1161	0.4100*	0.3890*	0.1102
Thaumarchaeota; Marine Group I	-0.4032*	-0.5658***	0.3844*	-0.1221	-0.3502*	-0.1818	0.1984	0.1795	0.0389
Thaumarchaeota; Soil Crenarchaeotic Group	-0.2955	-0.7094***	0.3299	-0.1218	-0.2855	-0.2221	0.0915	0.0731	-0.0126
Others	-0.1437	0.4578**	-0.2442	0.0909	0.1626	0.2640	0.1534	0.1671	-0.4377*

Correlation coefficient with *p*-value score less than 0.05, 0.01, 0.001 is labeled with "*", "**", "***", respectively.
